# Structures, activity and mechanism of the Type IIS restriction endonuclease PaqCI

**DOI:** 10.1093/nar/gkad228

**Published:** 2023-03-29

**Authors:** Madison A Kennedy, Christopher J Hosford, Caleigh M Azumaya, Yvette A Luyten, Minyong Chen, Richard D Morgan, Barry L Stoddard

**Affiliations:** Division of Basic Sciences, Fred Hutchinson Cancer Research Center, 1100 Fairview Ave. North, Seattle,WA 98109, USA; New England Biolabs, 240 County Road, Ipswich, MA 01938, USA; Division of Basic Sciences, Fred Hutchinson Cancer Research Center, 1100 Fairview Ave. North, Seattle,WA 98109, USA; New England Biolabs, 240 County Road, Ipswich, MA 01938, USA; New England Biolabs, 240 County Road, Ipswich, MA 01938, USA; New England Biolabs, 240 County Road, Ipswich, MA 01938, USA; Division of Basic Sciences, Fred Hutchinson Cancer Research Center, 1100 Fairview Ave. North, Seattle,WA 98109, USA

## Abstract

Type IIS restriction endonucleases contain separate DNA recognition and catalytic domains and cleave their substrates at well-defined distances outside their target sequences. They are employed in biotechnology for a variety of purposes, including the creation of gene-targeting zinc finger and TAL effector nucleases and DNA synthesis applications such as Golden Gate assembly. The most thoroughly studied Type IIS enzyme, FokI, has been shown to require multimerization and engagement with multiple DNA targets for optimal cleavage activity; however, details of how it or similar enzymes forms a DNA-bound reaction complex have not been described at atomic resolution. Here we describe biochemical analyses of DNA cleavage by the Type IIS PaqCI restriction endonuclease and a series of molecular structures in the presence and absence of multiple bound DNA targets. The enzyme displays a similar tetrameric organization of target recognition domains in the absence or presence of bound substrate, with a significant repositioning of endonuclease domains in a trapped DNA-bound complex that is poised to deliver the first of a series of double-strand breaks. PaqCI and FokI share similar structural mechanisms of DNA cleavage, but considerable differences in their domain organization and quaternary architecture, facilitating comparisons between distinct Type IIS enzymes.

## INTRODUCTION

Bacteria have evolved a wide variety of factors and pathways for antiviral defense. Systems that directly target foreign DNA for degradation or inhibition of its replication include both *innate* defense systems, such as restriction endonucleases and their cognate methyltransferases (restriction-modification or ‘RM’ enzymes), that rely on the protection of the host genome while simultaneously targeting incoming foreign DNA for degradation, (1–3) and *adaptive* defense systems typified by CRISPR-associated nucleases (4). Those two well-studied ‘front line’ microbial defenders are augmented by (i) a variety of additional defense systems that also target foreign DNA for degradation or replication interference, (ii) additional systems that trigger abortive infections and cell death, and (iii) many newly-discovered, uncharacterized factors that are encoded within mobile genetic defense islands and are believed to also play additional roles in combatting phage challenges (see (5) for a recent review).

RM systems are comprised of restriction endonuclease (‘REase’) and methyltransferase (‘MTase’) enzymes and activities that are coupled to one another genetically (and often structurally) (6–8). Such systems operate by searching for short target sequences found in foreign invading DNA duplexes and cleaving within or near those sites while chemically modifying and protecting the same sequences in host DNA (9,10). RM systems are historically classified into four main types, defined by their subunit architectures, target search mechanisms, cofactors, and catalytic behaviors (7,11). In Type I, II and III RM systems, unmodified foreign DNA targets are recognized and cleaved, while corresponding targets in the host DNA are modified to prevent cleavage of the host's genome. In contrast, type IV systems recognize and cleave foreign DNA targets that are proactively modified by phage as a countermeasure to avoid cleavage.

Type I, II and III RM systems differ primarily in their DNA cleavage mechanisms. Type I and III enzymes bind their specific target through a DNA recognition domain partnered with the methyltransferase and then require ATP-dependent translocase subunits to bring together multiple bound targets assembled into active enzyme complexes capable of DNA cleavage (1,3). In contrast, Type II enzymes are simpler and cleavage generally requires only magnesium as a cofactor (2). Type II systems most often consist of separate REase and MTase enzymes, each with their own DNA recognition domain targeted to the same sequence motif, although some forms rely upon a single, shared binding domain to target both activities to the same DNA sequence. The REase and MTase search for their target via localization near DNA, followed by rapid association and dissociation coupled with limited two-dimensional (2D) diffusion along the double helix. For many of the most familiar Type II enzymes, the REase is a homodimer that acts separately at each individual site with each monomer cutting one DNA strand.

Type II enzymes, which are most commonly employed for molecular cloning and biotechnology applications, are further distinguished and categorized by differences between (i) their cleavage patterns (cutting either within their targets or at a fixed distance to one (or both) sides of their bound targets), (ii) their catalytic behavior (sometimes displaying non-cooperative cleavage of a single bound target, but often relying upon allosteric or cooperative cleavage mechanisms that require the simultaneous engagement of multiple target sites for maximum cleavage efficiency), and (iii) their domain organization (displaying various arrangements of protein domains involved in target recognition, cleavage and/or methylation) (2,12,13). These distinctions lead to the division of Type II REases into multiple subtypes. This includes Type IIP enzymes (usually homodimers that cleave palindromic target sites), Type IIE and IIF (frequently multimeric complexes, often tetramers, that require binding of at least two target sites to cleave one site and that exhibit cooperative and/or allosteric behaviors during cleavage of those two sites), and Type IIT (heterodimers that cleave asymmetric sites) (12,14–16). In addition, more complex Type IIG and IIL enzymes contain both REase and MTase domains that are physically coupled and that act in a coordinated manner to either methylate or cleave self or nonself DNA, respectively (17).

Type IIS restriction endonucleases (the topic of this study) combine independently folded target recognition (‘TRD’) and endonuclease (‘EN’) domains within a single protein chain, cleaving DNA at one or both sides of their target site (18). They are of particular interest for biotechnology, being the source of nonspecific EN domains for various gene-targeting nuclease platforms (including zinc finger nucleases and TAL effector nucleases) (19) and also being used for a wide variety of genome mapping and gene assembly applications (20,21). Individual Type IIS enzymes display considerable variation in (i) their architecture (with the EN domain found at either the N- or C-terminal end of a protein subunit), (ii) the identity of the nuclease active site motif (usually containing HNH or PD-(D/E)xK active sites) and their cleavage mechanism, and (iii) their cleavage positions (cleaving at precise distances from the bound target, with those distances varying greatly between different enzymes) (22).

The most thoroughly studied Type IIS restriction endonuclease, FokI, recognizes a five-base, non-palindromic sequence (5′-GGATG-3′) and cuts the top and bottom DNA strands 9 and 13 bases downstream from its target site, generating a complementary four-base 5’ overhang (23). It is comprised of an N-terminal TRD and C-terminal EN domain that harbors a single PD-(D/E)xK catalytic motif (23). Crystal structures of inactive FokI in the absence or presence of bound DNA illustrate similar monomeric protein conformations, with the nuclease domain loosely docked against the TRD but not positioned appropriately for DNA cleavage (indicating that additional motion of that domain is required to form the eventual reaction complex) (23,24). Those results, along with detailed kinetic studies (25–29), have indicated that a multimeric assemblage of two DNA-bound enzyme subunits, along with the additional movement of the EN domains, is required for DNA cleavage. Additional studies using single-molecule force measurements (30,31) and electron microscopy (EM) imaging (32) further indicate a mechanism in which the formation of a dimeric enzyme-DNA cleavage synapse produces a parallel arrangement of bound DNA targets and significant DNA looping. However, a high-resolution view of the ultimate reaction synapse formed by FokI (or any other Type IIS enzyme) has not yet been described.

Here, we report biochemical analyses and high-resolution crystallographic and CryoEM structures (in the presence and absence of bound DNA) of the Type IIS REase PaqCI. While this enzyme is also a Type IIS enzyme with a PD-(D/E)xK active site motif; its domain architecture is reversed as compared to FokI (corresponding to an N-terminal EN domain). It forms a tetramer in solution in both the absence and presence of bound DNA, recognizes a longer target than FokI (5′-CACCTGC-3′), and cleaves each strand of bound DNA much closer to the bound target site (4 and 8 bases downstream, respectively). Our analysis, therefore, present a detailed examination of an alternative, but related Type IIS enzyme while also providing a high-resolution visualization of the conformational changes that accompany DNA binding and of the enzyme trapped in a catalytically productive conformation, poised to deliver the first in a series of DNA cleavage events. Our structure explains why Type IIS enzymes such as PaqCI require binding at multiple sites to activate cleavage. The study of PaqCI provides an important basis for comparison, analysis, and additional understanding of the diversification and action of Type IIS enzymes.

## MATERIALS AND METHODS

### Enzyme identification, cloning, expression and purification

DNA encoding the *Paucibacter aquatile* Type IIS restriction endonuclease (REase), PaqCI, was codon-optimized for *Escherichia coli* expression and synthesized commercially by Integrated DNA Technologies (IDT, Coralville, IA) and cloned into a modified pACYC184 T7 expression vector. DNA encoding the full-length BspMI methyltransferase, M.BspMI, was cloned into a modified pBR322 expression vector. Untagged PaqCI/pACYC184-T7 and M.BspMI/pBR322 were co-transformed into T7 Express Competent *E. coli* (NEB), grown at 37°C in Terrific Broth to an *A*_600_ of 1.0, and then induced with 0.3 mM isopropyl 1-thio-β-d-galactopyranoside (IPTG) overnight at 19°C. Cells were harvested by centrifugation, washed with DEAE load buffer (20 mM Tris–HCl, pH 8.5, 300 mM sodium chloride, 5 mM β-mercaptoethanol), and pelleted a second time. Pellets were flash-frozen in liquid nitrogen and stored at −80°C.

Thawed pellets from 2 l cultures were resuspended in 100 ml of DEAE load buffer supplemented with 10 mM phenylmethylsulfonyl fluoride (PMSF), 5 mg of recombinant DNaseI (NEB), and 5 mM magnesium chloride. Lysozyme was added to 1 mg/ml, and the mixture was incubated for 15 min rocking at 4°C. The cells were disrupted by sonication, and the lysate was cleared of debris by centrifugation at 13 000 rpm (19 685 × g) for 30 min at 4°C. The supernatant was sterilized through a 0.45 μm syringe filter, loaded onto 2×5 mL HiTrap DEAE columns, and flowthrough fractionated. Fractions were assayed for PaqCI endonuclease activity. Peak fractions were analyzed by SDS-PAGE, pooled, and dialyzed against Heparin load buffer (20 mM HEPES, pH 7.5, 50 mM sodium chloride, 5% glycerol, 1 mM EDTA, and 1 mM DTT). The sample was loaded onto 2×5 ml HiTrap Heparin columns, washed with load buffer, eluted in a gradient from 50 mM to 500 mM sodium chloride, and fractionated. Peak fractions were analyzed by SDS-PAGE, pooled, concentrated, and further purified by size exclusion chromatography (SEC) using a Superdex 200 10/300 GL column. The sample was exchanged into a final buffer of 20 mM HEPES, pH 7.5, 150 mM sodium chloride, 5 mM magnesium chloride, and 1 mM DTT during SEC and concentrated to 10-20 mg/ml (Supplementary Figure S1).

### Biochemical analyses of target site specificity and cleavage activity

Oligoduplex primers (Supplementary Table S1) were synthesized by IDT. Molecular biology reagents including Q5 Hot Start High-Fidelity DNA polymerase, NEBuilder HiFi DNA assembly master mix, restriction enzymes, DNA size standards, lambda DNA, and competent cells were from New England Biolabs (NEB). Plasmid purification and nucleic acid purification clean ups were performed using Monarch DNA kits (NEB). Plasmid DNA constructs were confirmed by sequencing on an ABI 3130xl capillary machine (Applied Biosystems).

#### PaqCI-site plasmid substrate construction

Plasmid DNA substrates were constructed containing either a single PaqCI recognition site, or two recognition sites in either head-to-head (HTH) or head-to-tail (HTT) orientation, or with 4 recognition sites arranged in two different formats. PaqCI recognition sites were introduced at position 1 and/or position 700 of pUC19 by mutagenic PCR. The single-site construct (p1SS) was PCR amplified using PaqCI_pUC19 1 For/PaqCI_pUC19 1 Rev (see Supplementary Table S1) primer pairs. Two-site constructs (p700HTT and p700HTH) were amplified as two amplicons: PaqCI_pUC19 1For/PaqCI_pUC19 700HTT Rev & PaqCI_pUC19 700HTT For/PaqCI_pUC19 1 Rev or PaqCI_pUC19 1 For/PaqCI_pUC19 HTH Rev & PaqCI_pUC19 700HTH For/PaqCI_pUC19 1 Rev (see Supplementary Table S1). PCR amplicons were confirmed by agarose gel electrophoresis. Template DNA was removed by digestion with DpnI (NEB) at 37°C for 30 min and purified using NEB Monarch nucleic acid purification kit following manufacturer's instructions. Purified amplicons were assembled using NEBuilder® HiFi DNA assembly and transformed into NEB® 5α chemically competent *E. coli* according to manufacturer's instructions. Individual colonies were grown overnight in LB broth supplemented with ampicillin (100 μg/ml). Two unique four-site plasmid DNA substrates were created by inserting an approximately 850bp fragment containing the chloramphenicol gene amplified from pACYC184 flanked on both ends by PaqCI recognition sites into either the HTH or HTT 2-site plasmid substrates described above. The additional DNA was inserted 800bp downstream of the second recognition site using NEBuilder HiFi DNA assembly master mix following manufacturer's instructions. Plasmid DNAs were isolated from overnight cultures, and the introduced PaqCI recognition sites were confirmed via Sanger sequencing.

#### PaqCI cleavage activity

One unit of enzyme is defined as the amount of PaqCI required to digest 1 μg of lambda phage DNA to completion in 1 h at 37°C in a 50 μl volume. One unit of PaqCI is equivalent to 24 ng or 0.43 picomoles of enzyme monomer, which in a 50 μl reaction corresponds to 8.6 nM enzyme monomer concentration. The concentration of target sites in lambda phage DNA (containing 12 PaqCI sites) in the same 50 μl reaction is 7.2 nM. Digests were performed either with or without a *trans*-activating oligonucleotide (a short double-stranded hairpin DNA construct spanning the PaqCI recognition site, corresponding to the sequence 5’- GGA GCAGGTG AGCGAG TTTT CTCGCT CACCTGC TCC-3’, that possesses the binding site (underlined) and does not extend past the point of cutting). The range of enzyme concentrations employed in units (16–0.125 units) corresponds to approximately 140–1 nM enzyme monomer. For clarity, we report enzyme monomer and DNA target sites concentrations because each enzyme monomer will bind one DNA target site, while acknowledging that in solution the enzyme exists as a tetramer that can bind up to four target sites simultaneously.

The single-site plasmid substrate (p1SS), dual-site plasmid substrates (p700HTT and p700HTH), and lambda DNA (NEB) were digested with variable concentrations (140–2.2 nM) of PaqCI, either in the presence or absence of the *in trans*-activating DNA oligoduplex. PaqCI enzyme was serially diluted in reaction buffer (rCutSmart™: 20 mM Tris-acetate, 10 mM magnesium acetate, 50 mM potassium acetate, 100 μg/ml recombinant albumin, pH 7.9 @ 25 °C) containing 1 μg substrate DNA per 50 μl and incubated for 1 h at 37 °C. Reactions with *trans*-activating oligoduplex were performed identically, with the activator added to the first reaction at a concentration of 40 nM activator per unit (24 ng or 8.6 nM) of PaqCI and then serially diluted with the enzyme to maintain a constant ratio of approximately 5:1 activator to enzyme. Digestion reactions were visualized on a 1% agarose gel.

The relative rate of REase activity and whether sites are cleaved in a coordinated manner was examined by cleavage of the dual-site plasmids p700HTT and p700HTH, either in the presence and absence of activating oligoduplex. Cleavage assays were performed at 37°C in rCutSmart™ buffer containing 1 μg substrate DNA (22.8 nM PaqCI sites), 2 units (48 ng or 17.2 nM) of PaqCI per 1 μg of DNA, and 80 nM activating oligoduplex (40 nM per unit of PaqCI) when indicated. Aliquots of the reaction were removed at 0.25 min, 0.5 min, 1 min, 3 min, 5 min, 10 min, 30 min and 1 h. Reactions were terminated by adding stop solution containing 0.08% SDS (NEB Gel Loading Dye, Purple), and digestion reactions were visualized on a 1% agarose gel.

Substrates containing four target sites were digested using a ratio of 0.5:1, 1:1, 2:1 and 4:1 enzyme-binding-domains to DNA-recognition-sites ratio over a time course. To examine cleavage kinetics the 4-site plasmid was pre-bound with enzyme at a 1:1 enzyme-binding-domain to recognition-site ratio in a buffer equal to CutSmart buffer but lacking any Mg^2+^ ions required for DNA cleavage to allow the DNA-enzyme complex to form prior to initiating cutting. Pre-binding was performed for 15 min at 37°C in rCutSmart™ buffer lacking Mg^2+^ ions containing 1 μg substrate DNA in 50 μl (37 nM PaqCI sites) and 4.3 units (37 nM) of PaqCI. Cleavage was initiated by adding Mg^2+^ to 10 mM and aliquots were removed at 0.25 min, 0.5 min, 1 min, 3 min, 5 min, 10 min, 30 min and 1 h.

### X-ray crystallographic analyses

Crystals of the DNA-free PaqCI apo-enzyme (Supplementary Figure S2) were grown using protein purified as described above. Protein samples dispensed in 1 μl drops (at concentrations ranging from 3 to 12 mg/ml in 20 mM HEPES pH 7.5, 150 mM potassium chloride, 5% glycerol v/v) were mixed with an equal volume of a crystallization solution containing 20 to 25% w/v polyethylene glycol (PEG) 3350, 0.2 M magnesium chloride hexahydrate and 0.1 M Bis–Tris pH 5.5. Vapor phase equilibration of the resulting drops against a 1 ml reservoir of the same crystallization solution resulted in the growth of crystals with dimensions of 0.05–0.2 mm in each dimension within approximately 72 h. The crystals were flash cooled in liquid nitrogen after transfer into a cryoprotective solution corresponding to elevated PEG 3350 (30% w/v) and 20% ethylene glycol. Diffraction data were collected on a Pilatus areas detector at the Advanced Light Source (ALS) synchrotron facility at beamline 5.0.1. The resulting data set (Table 1) extended to 2.5 Å resolution and corresponded to a primitive tetragonal space group (*P*4_3_2_1_1) in which two copies of the protein subunit occupied the asymmetric unit.

**Table 1.** Crystallographic data and refinement statistics

**Data statistics**	
Space group	*P*4_3_2_1_2
Wavelength(s)	0.9762 (Å)
Unit cell dimensions	*a* = *b* = 136.6 Å, *c* = 106.4 Å
Resolution	2.5 Å (2.54–2.5)
Reflections	35387
Completeness	100.0% (100.0)
Redundancy	26.1 (25.9)
I/σ(I)	43.9 (5.6)
R_merge_	0.0691 (0.422)
R_pim_	0.017 (0.095)
CC_1/2_	1.000 (0.995)
**Refinement statistics**	
PDB ID	8EM1
Resolution	2.5 Å
No. reflections	35 124 (3427)
*R*_work_/*R*_free_	0.2089/0.2641
No. atoms	
Protein	973
Ligand/Ion	8
Water	69
*B*-factors	
Protein	55.46
Ligand/ion	57.67
Water	47.22
RMS deviations	
Bond length (Å)	0.009
Bond angles (°)	1.04

Data was processed using program HKL2000 (33). The placement of two copies each of the N-terminal endonuclease (EN) domain and the C-terminal target recognition domain (TRD) into the asymmetric unit was then performed using the molecular replacement algorithm in program PHENIX (34). The molecular replacement searches were conducted in two sequential steps, using independent molecular models for the EN and TRD domains that were each generated using the program AlphaFold (35). An initial search with the model of the N-terminal domain produced a solution with excellent signal (LLG = 177.6; TFZ = 12.1) that placed two EN domains in a dimeric arrangement that appeared appropriate for eventual engagement with a DNA duplex. After fixing those domains, a second search then placed two copies of the model of the C-terminal TRD into the remaining volume of the asymmetric unit built, resulting both in strong signal (LLG 317.5; TFZ = 9.2) and distances between their termini that could easily be bridged by the peptide linker sequence connecting the two domains.

Local rebuilding of the two enzyme subunits in the asymmetric unit, including the modeling of a peptide linker in each that connects an N-terminal EN domain to its corresponding C-terminal domain) was performed using the program COOT (36), followed by refinement using the program PHENIX (34). The final values for *R*_work_/*R*_free_ were 0.21/0.26 with good geometry (Table 1).

The complete structure of the DNA-free apo-enzyme was ultimately found to correspond to a compact tetrameric assemblage, in which two additional protein subunits are generated via application of a crystallographic dyad symmetry axis. The designation of the enzyme assemblage as a tetramer agrees with (i) the solution behavior of the protein when analyzed and purified via size exclusion chromatography, and (ii) the same structure determined independently via single-particle cryogenic electron microscopy (CryoEM), as described below.

### CryoEM sample preparation and data collection

All samples for PaqCI DNA-free and DNA-bound CryoEM complexes were generated using purified protein as described above. The sample (with/without DNA) was filtered through a 0.22 μm centrifugal filter and loaded onto a HiLoad 16/600 Superdex 200 prep grade size exclusion column (Millipore Sigma) equilibrated in 30 mM BisTris pH 6.5, and 100 mM sodium chloride to assess oligomerization state. The DNA-bound PaqCI complex was generated by incubating enzyme and DNA in excess at a 1:1.2 molar ratio (each monomer possessing one DNA binding site). This was done in the presence of 10 mM calcium chloride to prevent cleavage of the DNA substrate. The protein co-eluted with the DNA generating a peak centered at an estimated molecular weight of approximately 225 kD with no remnants of an unbound peak (Supplementary Figure S3a). The oligomer peak was taken from the SEC and diluted. Each complex was initially evaluated via negative stain electron microscopy (EM) for optimization of CryoEM sample preparation (Supplementary Figures S2 and S3). Negative stain grids were prepared by adding the sample to glow-discharged uniform carbon film coated grids and stained with 0.75% uranyl formate as in Ohi, 2004 (37). Data was collected semi-automatically with Leginon (38) using the Fred Hutchinson Cancer Center's 120 kV ThermoFisher Talos 120C LaB6 microscope equipped with a Ceta camera.

Vitrification conditions for CryoEM grids were screened with the optimized enzyme sample from negative stain EM. CryoEM grids were prepared by applying 2 to 4 μl of complex to a glow-discharged Quantifoil R1.2/1.3 or UltrAuFoil R1.2/1.3 300 mesh grid, which was blotted for 2–8 s, then plunge frozen in liquid ethane using an FEI Vitrobot Mark IV (ThermoFisher) at 4°C and 100% humidity. All data sets were collected using the Fred Hutchinson Cancer Center's 200 kV ThermoFisher Glacios X-FEG electron microscope equipped with a Gatan K3 detector. SerialEM was used for data acquisition (39). Six second movies were collected at 0.06 s per frame with a per frame dose of 0.5 e^–^/Å2/s. The pixel size on the specimen was 0.561 Å/pixel. Collection and on-the-fly monitoring were conducted with Warp for ctf estimation, motion correction, and data quality monitoring (40).

To generate a map of the unbound structure, three 0° tilt and one 40° tilt data sets were collected. The first two 0° tilted CryoEM data sets for the unbound structure were collected using 0.1 mg/ml and 0.4 mg/ml complex, respectively, on Quantifoil R1.2/1.3 grids with 4 second blot times. 869 movies were collected for the first data collection, and 2,075 movies were collected for the second data collection. The final datasets for the unbound structure were collected using an UltrAuFoil 1.2/1.3 grid with 0.26 mg/ml protein blotted for 6 s. 276 movies were collected at 0° tilt and 1325 movies were collected at 40° tilt. Two data sets were collected for the DNA-bound PaqCI structure, one at 0° and one at 40° tilt that were collected on an UltrAuFoil R1.2/1.3 grid with 0.9 mg/ml complex blotted for 6 s. 941 movies were collected at 0° tilt, and 956 movies were collected at the 40° tilt.

### Negative stain data processing

Micrographs were imported into RELION 3.1 (41) and ctf corrected using CTFFIND4 (42). Particles were manually picked to establish two-dimensional (2D) templates and then template picked. These particles were extracted in a 300-pixel box and binned by 4 before extraction and 2D classification to assess particle homogeneity.

### CryoEM data processing

Motion-corrected data that had been binned by two in WARP was downloaded and imported into cryoSPARC (43) for unbound and DNA-bound structures. Once in cryoSPARC, ‘patch ctf’ was run (43). Each independent dataset was preliminarily processed before combining after initial particle curation. The preliminary processing for both structures included ctf estimation, exposure curation, blob picking (to generate 2D templates), template picking, 2D classification, ab-initio reconstruction, and heterogeneous refinement. Templates were selected from the first round of 2D classification and used for future particle picking. Data was refined into three classes using heterogeneous refinement. One good class was selected and moved on to the next round of refinement. The process was iterated three times. Box size remained at 64 pixels for the initial 2D classifications, then expanded to 128 pixels for the heterogeneous refinement. All data sets for each structure were combined after the final heterogeneous refinement and further processed with an additional round of 2D classification, and iterative non-uniform 3D refinement. Data was unbinned once all data sets were combined and one round of 2D classification was performed and particles were extracted with a 300-pixel box size. Before generation of the final map, global ctf and local ctf jobs were run. The final selected particles were run through non-uniform refinement to generate the final 3D CryoEM map. Local resolution was determined using the final non-uniform refinement job. No symmetry was ever imposed during data processing of either complex. A flowchart of the processing can be found in Supplementary Figure S4a.

For the unbound PaqCI, a total of 2 118 800 putative particles were initially picked from the four data collections after template picking. In the final structure, approximately 982 000 particles were used from the 0° tilt datasets, and approximately 420 000 particles were used from the 40° tilt dataset. After processing using the pipeline described above, a total of 1 427 503 particles were used to generate a 3.0 Å (global resolution) map, which was subsequently used to confirm the tetrameric structure seen in crystallography.

For the DNA-bound PaqCI, a total of 299 211 putative particles were template-picked from the two data collections. After processing using the pipeline described above, a total of 166 130 particles were used to generate a 3.15 Å (global resolution map). Additional methodological details of the CryoEM analysis of the DNA-bound PaqCI and a corresponding workflow chart are provided in Supplementary Figure S4.

### CryoEM model building and refinement

The refined X-ray crystallographic model of unbound PaqCI were docked into CryoEM maps, confirming the architecture of the DNA-free enzyme tetramer determined as described above. The structure of the DNA-bound PaqCI was built and refined using COOT (36), UCSF Chimera (44), and Phenix (34). Fitting of the atomic model (from unbound PaqCI crystal structure) into the CryoEM map was performed using UCSF Chimera (44) and was manually adjusted in COOT (36). First, the tetrameric TRDs were docked into density, and then the two visible EN domains were docked in the remaining densities. Once the oligomer was assembled, it was ported into Phenix for refinement using the phenix.real_space_refine application (34). After a few rounds of refinement, DNA oligos were added to the complex one at a time, with a round of refinement in between each addition. The final model for DNA-bound PaqCI was refined in Phenix with secondary structure and geometry restraints (Table 2). All figures and morphs were generated with PyMOL, and all movies were generated with UCSF Chimera (44).

**Table 2.** CryoEM data collection, refinement and validation statistics

Data collection	
EM equipment	Glacios X-FEG (Thermo Fisher)
Voltage (kV)	200
Detector	Gatan K3
Pixel size (Å/pixel )	0.561
Electron dose (e^-^/Å^2^)	50
Defocus range (μm)	0.1–4.0
Number of collected micrographs	1897
Number of used micrographs	1397
**Reconstruction**	
Software	cryoSPARC, RELION
Number of used particles	166,130
Symmetry	No symmetry
Resolution (Å)	3.15
Map sharpening *B*-factor (Å^2^)	139.1
**Refinement**	
PDB/EMD ID	8EPX/EMD-28534
Software	Phenix
Cell dimensions	
*a*=*b*=*c* (Å)	336.6
α=β=γ (°)	90
Model composition	
Protein residues	1663
Side chains assigned	1663
MolProbity score	2.27
Rms deviations	
Bonds length (Å)	0.003
Bonds angle (°)	0.530
Ramachandran plot statistics (%)	
Most favored	94.82
Allowed	5.18
Outlier	0.00

## RESULTS

### Purification and biochemical characterization

The purified enzyme runs as a large single band in an SDS-PAGE gel, with a larger faint band that may correspond to a larger persistent oligomer (Supplementary Figure S1a). The same protein sample behaves as a multi-subunit assemblage (appearing to correspond to a protein tetramer) when eluting from a size exclusion chromatography column (SEC) (Supplementary Figure S1b).

**Figure 1.**
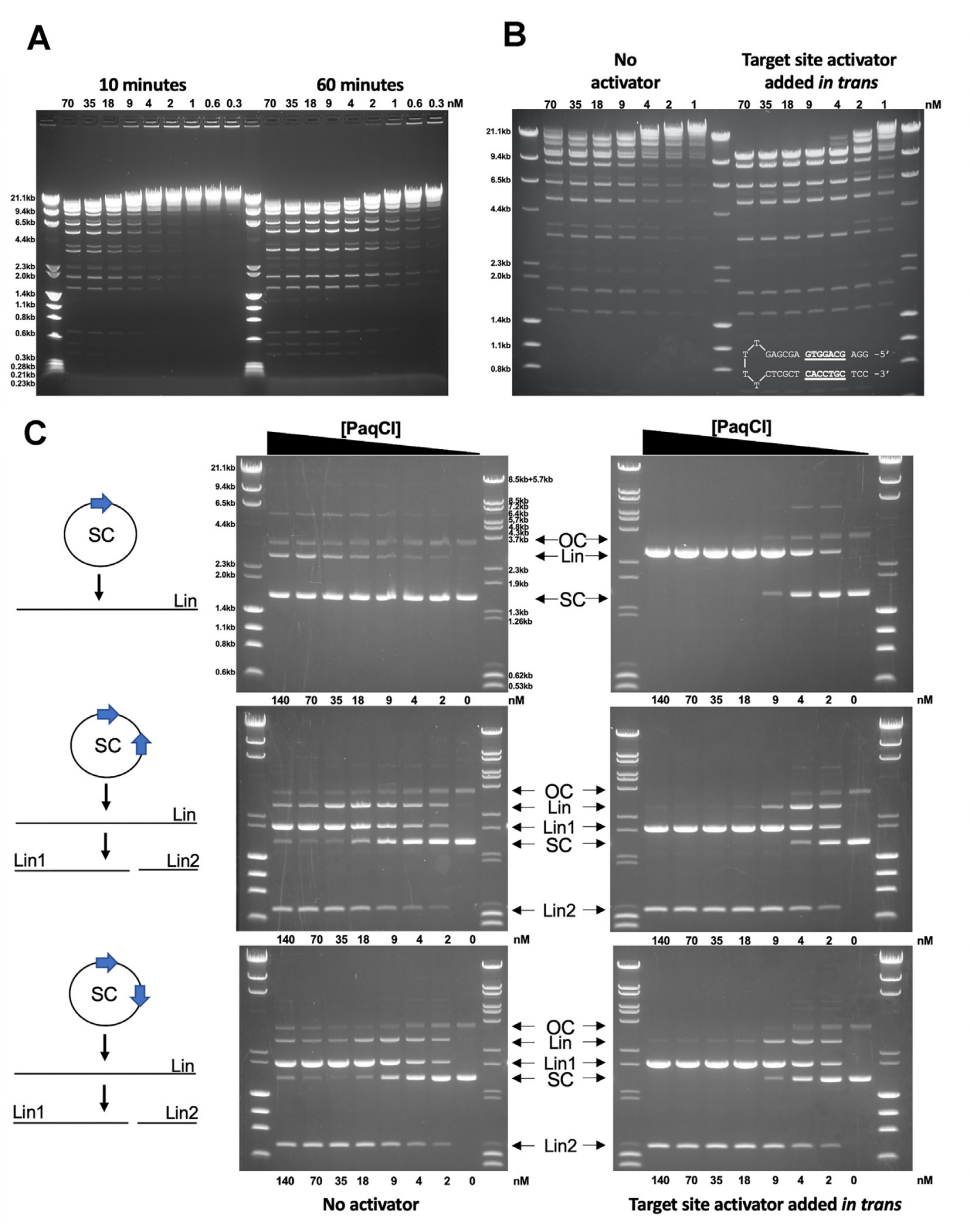
Cleavage activity of PaqCI towards lambda phage DNA and plasmid substrates. (**A**) PaqCI demonstrates optimal activity at an equal to modest excess of enzyme binding sites to substrate target motifs, with an excess of enzyme to substrate leading to faster, but not more complete, cutting. Lambda phage DNA, with 12 PaqCI sites, are digested and quenched at various time points. Time points correspond to 10- and 60-min digests, with the concentration of enzyme monomers varied from 0.3 to 70 nM. The DNA recognition site concentration in all digests was 7.2 nM. At 60 min, enzyme concentrations greater than 1:1 enzyme-to-sites exhibit near-complete cleavage, while 0.5:1 enzyme-to-sites generates only slightly less cutting, and lesser amounts of enzyme result in more partial digestion. The molecular weight ladders Lambda-HindIII/PhiX174_HaeIII were used in panel a and b which spans 0.23 to 23.2 kb. This experiment was performed once. Concentrations are rounded to the nearest decimal place. (**B**) PaqCI cleaves DNA more efficiently when a short hairpin DNA duplex (inset in right gel panel of B) containing the enzyme's target site (underlined bases), is added to the reaction as a *trans*-activating factor. Lambda phage DNA was digested with variable concentrations of PaqCI from 1 to 70 nM (monomeric concentration) in the absence or presence of the *trans* activator (which was added at a 5:1 ratio to the PaqCI enzyme, i.e. 350 to 5 nM activator). All lanes correspond to 60-min digests. This experiment was performed once. Units were rounded to the nearest decimal place. (**C**) Further experiments examine the cleavage of plasmid substrates and demonstrate that PaqCI requires the interaction between multiple bound sites for cleavage. All lanes correspond to 60-min digests at fixed DNA concentrations, with variable enzyme (monomeric concentrations). The single-site substrate (11.4 nM sites) is cleaved far less efficiently than the 2-site substrates (22.8 nM sites). The difference in cleavage efficiency between single- or multiple-target plasmid substrates is eliminated by the presence of the activator DNA (5:1 ratio to enzyme) containing a target site added in *trans*. The key shows open circle (OC), single-cut linear (Lin), double-cut products (Lin1, Lin2), and super-coiled (SC) species. Two molecular weight ladders were used in these experiments: (i) Lambda-HindIII/PhiX174_HaeIII which spans 0.6 kb to 23.1 kb, and (ii) Lambda-BstEII/pBR322-MspII which spans 0.5–8.5 kb. This experiment was performed once.

PaqCI demonstrates optimal activity against lambda phage DNA at an equal to modest excess of enzyme binding sites to substrate target motifs, with an excess of enzyme to substrate leading to faster, but not more complete, cutting. (Figure 1A). Digest time points are quenched at 10 and 60 min with protein (monomer) concentration ranging from 0.28 to 70.0 nM, while the DNA recognition site concentration is constant at 7.2 nM in each reaction. In the 10-min digest, near-complete cleavage of the DNA is only seen in concentrations from 17.5 to 70.0 nM, with partial cleavage to no cleavage in the rest of the lower concentrations. At 60 min, enzyme concentrations greater than 1:1 enzyme-to-sites exhibit near-complete cleavage, while 0.5:1 enzyme-to-sites generates only slightly less cutting, while lesser amounts of enzyme result in more partial digestion.

PaqCI cleaves lambda phage DNA more efficiently when a short hairpin DNA duplex containing the enzyme's target site (but not downstream DNA basepairs corresponding to the adjacent cleavage site) is added to the reaction as a *trans*-activating factor (Figure 1B). In this experiment, lambda phage DNA, with 12 PaqCI sites representing 7.2 nM sites, was digested with PaqCI from 70 to 1 nM in the absence or presence of the *in trans* target site oligoduplex, added at a 5:1 ratio to the PaqCI enzyme (350–5 nM activator). The presence of the *in trans* duplex target site enables essentially complete cutting of the substrate at enzyme concentrations greater than the concentration of substrate sites.

An additional experiment examining the cleavage of plasmid substrates (Figure 1C demonstrates that plasmid substrates containing two target sites are cleaved more efficiently than the same plasmid containing only one target site. The single-site substrate (11.4 nM sites) is only slightly cleaved while the 2-site substrate (22.8 nM sites) is nearly completely cut. The difference in cleavage efficiency between single- or multiple-target plasmid substrates is eliminated by the presence of the activator DNA (5:1 ratio to enzyme) containing a target site added in *trans*. The enzyme's cleavage efficiency against plasmids harboring two target sites does not appear to differ significantly when the target sites (which are separated by approximately 700 bp) are positioned in a head-to-head (HTH) or head-to-tail (HTT) arrangement.

**Figure 2.**
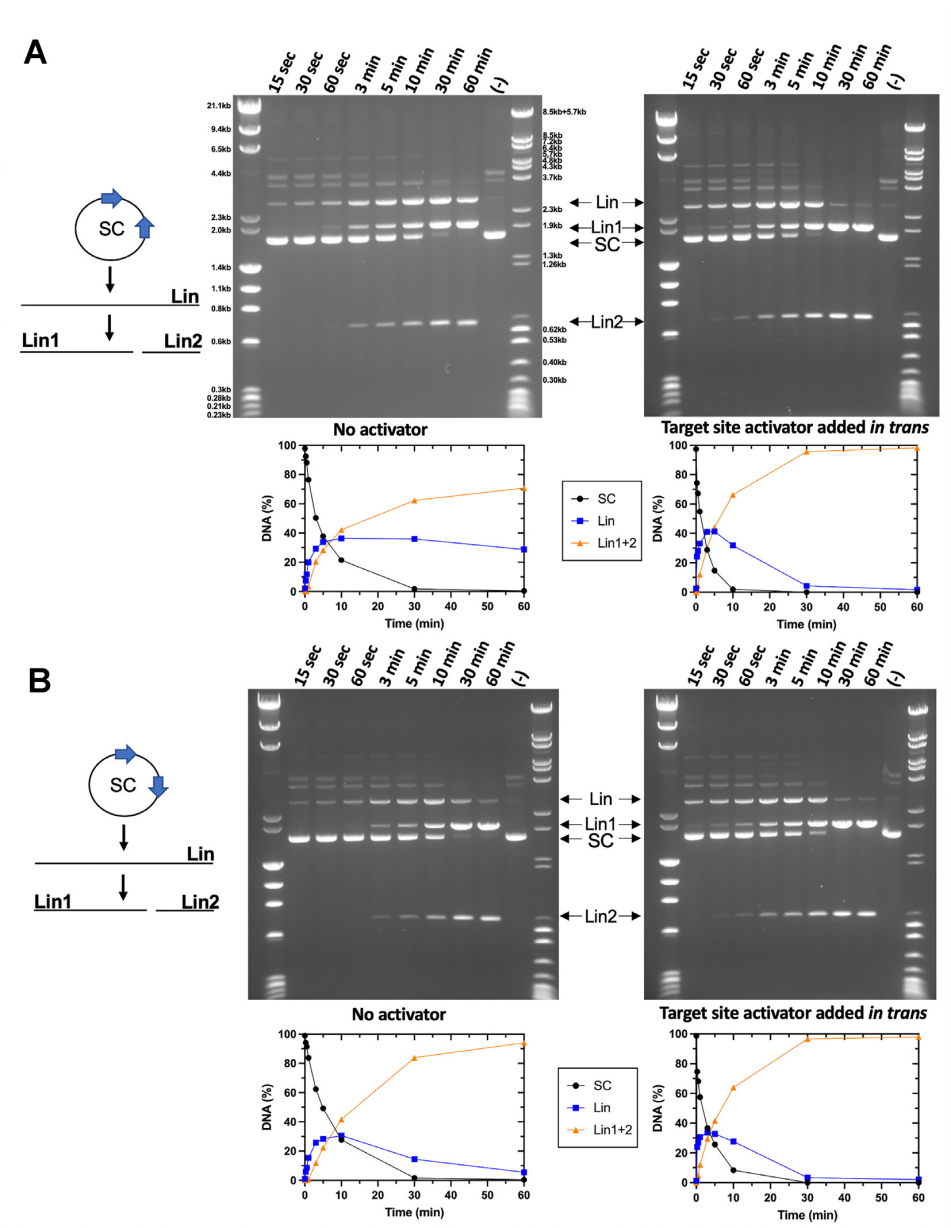
Time-dependent digestion of plasmids with 2 PaqCI target sites with and without added activator indicate a sequential cleavage profile. (**A**) The no activator reaction (left gel) contained 22.8 nM DNA target sites and 17.2 nM of monomer PaqCI per 22.8nM DNA sites incubated in 50 μl buffer. The activator reaction (right gel) contained the same proportion of DNA to PaqCI with 80 nM of added activating oligoduplex (4.7:1 activator to PaqCI monomer) incubated in 50 μl buffer. To the left of the gel is a cartoon of the head-to-head substrate and reaction products shown in the gel. Digest time points were quenched at 0.25, 0.5, 1, 3, 5, 10, 30 and 60 min. The mobilities of the super coil (SC), and the cleaved linear (Lin, Lin1, and Lin2) forms of the plasmid are indicated in the middle key with arrows corresponding to their related bands. The intensities of each substrate and product band in the gels were quantitated using ImageJ (54) software and calculated as rough percentages to be shown graphically. The key in the middle shows supercoiled DNA substrate (purple circles, SC), linearized DNA (blue square, Lin), and double cut long and short linearized DNA (orange triangle, Lin1+2). Unlabeled bands exist in the control (time 0) lane before PaqCI begins cleaving and may correspond to a species of multimeric plasmid. The bands are degraded as the experiment progresses, but no new species are made. They are not necessary to the conclusion of the experiments and were left unlabeled for clarity of the visual and are not included in the quantitation. Two molecular weight ladders were used in this experiment; (i) Lambda-HindIII/PhiX174_HaeIII which spans 0.2–23.1 kb, and (ii) Lambda-BstEII/pBR322-MspII which spans 0.3–8.5 kb. This experiment was performed once. (**B**) Head-to-tail reactions were quenched at the time points indicated above each lane and experiments were conducted as in Panel A. This experiment was performed once.

An additional experiment that measured a time course of plasmid substrate cleavage (Figure 2) demonstrated the rapid accumulation of an initial linearized product (‘Lin’ in the figure, corresponding to formation of a single double-strand break) followed by subsequent, slower generation of the final products corresponding to a second double-strand break (‘Lin1’ and ‘Lin2’ in the figure). In this experiment, relaxed or nicked circular plasmid does not visibly accumulate at the early points of the time course. When the same activator DNA construct harboring the PaqCI target site is added in *trans*, similar results are observed but occur on a faster time scale.

### Structure of the DNA-free enzyme

**Figure 3.**
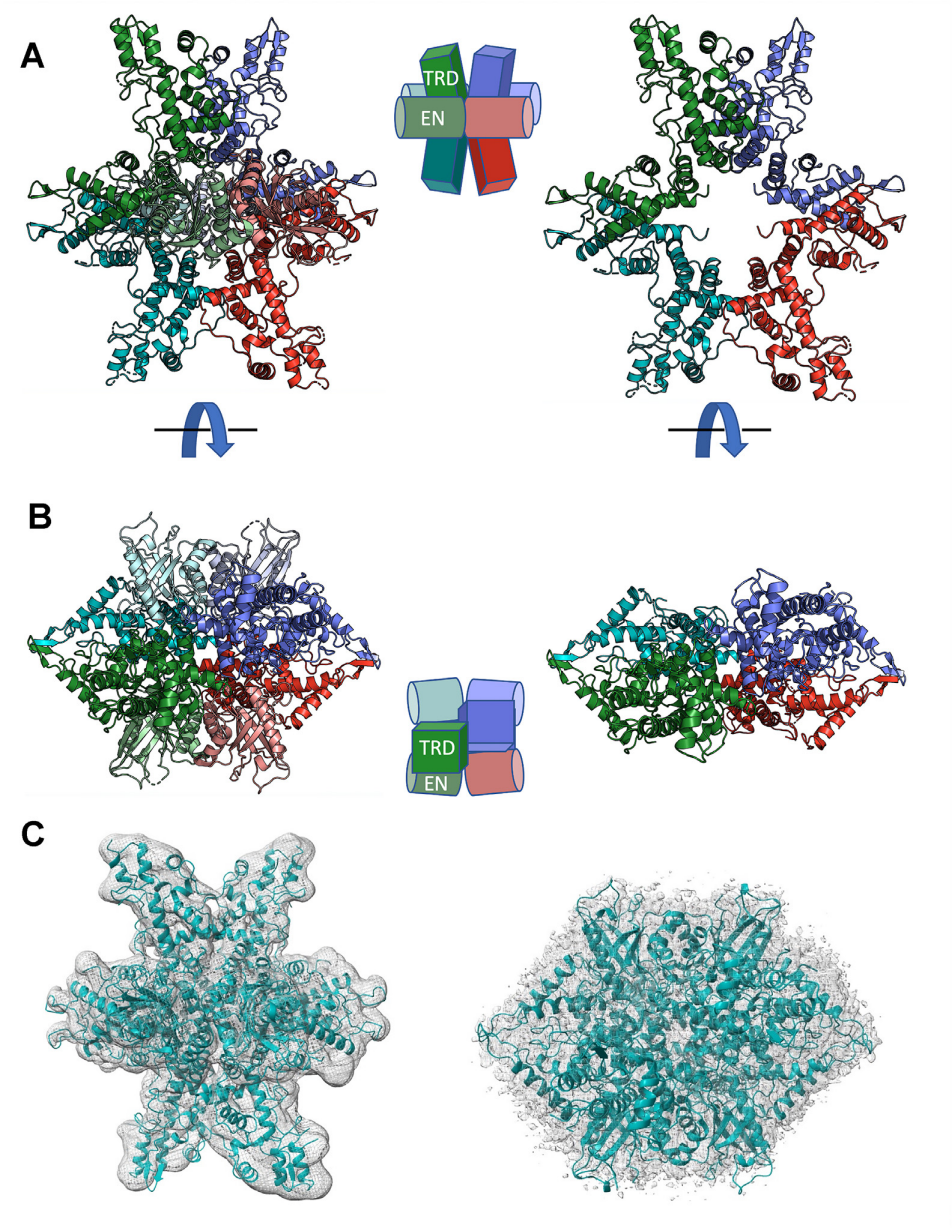
Crystallographic structure and CryoEM analysis of DNA-free PaqCI apo-enzyme. (**A**, **B**) Ribbon diagrams and cartoon representation of DNA-free apo-enzyme structure and domain organization. Each of the four protein subunits are colored independently and similarly in the two depictions. The endonuclease (EN) domains are colored in a lighter shade than the target recognition domains (TRDs) they are associated with. In the cartoons, the TRDs are represented by rectangles and the EN domains are shown as cylinders. The ribbon diagrams on the left illustrate the full-length protein subunits. The ribbon diagrams on the right illustrate only the C-terminal TRDs; the N-terminal EN domains are removed for clarity. The ribbon diagrams in Panel b are turned 90° around the x-axis when compared with Panel A. (**C**) Independently generated CryoEM electron density map, at approximately 3.0 Å global resolution, of the DNA-free apo-enzyme closely match the crystallographic model of the enzyme tetramer, validating the quaternary structure and domain organization in a solution-based analysis free of crystallographic contacts and lattice artifacts. The views seen in Panel c are the same orientations as Panels A and B. Crystals, diffraction quality, additional structural features, and representative negative stain EM and CryoEM micrographs and class averages are further illustrated in Supplementary Figure S2.

In the absence of bound DNA, PaqCI readily forms well-ordered diffracting crystals (Supplementary Figure S2a), implying that the DNA-free apo-enzyme forms a conformationally homogenous multimeric assemblage allowing it to be captured in a uniform crystal lattice. The crystal structure of the DNA-free enzyme was determined to 2.5 Å resolution (Table 1) and found to correspond to a compact enzyme tetramer (Figure 3A, B). In that structure, the asymmetric unit corresponds to an enzyme dimer, with a larger tetrameric assemblage generated via the application of a two-fold crystallographic symmetry axis, forming a dimer-of-dimers. The overall dimensions of the tetramer are 124 Å × 118 Å × 96 Å.

In the crystallographic model, four target recognition domains (TRD) and four endonuclease (EN) domains form a starburst-like structure. The TRDs form a ring of four C-shaped subunits while two EN domains dimerize on either side of the TRD ring, sequestering their active sites against the oligomer and away from solution and DNA. Each dimer pair within the enzyme tetramer contacts the other both through contacts between the sequestered EN domains and through additional contacts between the TRDs. The conformation of each individual protein subunit brings their N- and C-termini close to one another. Across the surface of the resulting tetrameric assemblage, basic residues are somewhat sparsely distributed, corresponding to a somewhat lower estimated p*I* (approximately 8.0) than is typically observed for many DNA binding proteins (Supplementary Figure S2b).

Unbound PaqCI forms visible particles in negative stain EM and CryoEM images (Supplementary Figure S2c, d), allowing an additional visualization of the structure of the unbound enzyme assemblage using single-particle cryogenic electron microscopy (CryoEM) and thereby validating (in solution and at a much lower protein concentration) the size, shape, and structural features of the DNA-free apo-enzyme described above. The sample displayed a limited set of preferred orientations in both negative stain EM and CryoEM requiring the need to tilt the stage to optimize visualization of the three-dimensional structure of tetramer. The final CryoEM map for the unbound enzyme, corresponding to approximately 2.9×3.3 Å resolution, was generated from 4284 micrographs and over 1 million particles. The refined crystallographic coordinates of PaqCI described above are easily docked into the CryoEM map with minimal rebuilding (Figure 3C).

3D variability analysis of the CryoEM DNA-free apo-enzyme tetramer map shows an oscillation between either side of the dimer pair (Supplementary Movie S1), with correlated motions of each TRD affecting the corresponding orientation of its neighbor. This correlated motion centered across the interface appears to illustrate that the unbound tetramer samples have slightly asymmetric conformations between the two dimer pairs. In each subpopulation, the EN domains remain associated with the tetrameric core. The visualized motion agrees with the observation that the lowest resolution of the CryoEM map corresponds to the ends of the TRDs (Supplementary Figure S2e).

### Structure of the DNA-bound enzyme

**Figure 4.**
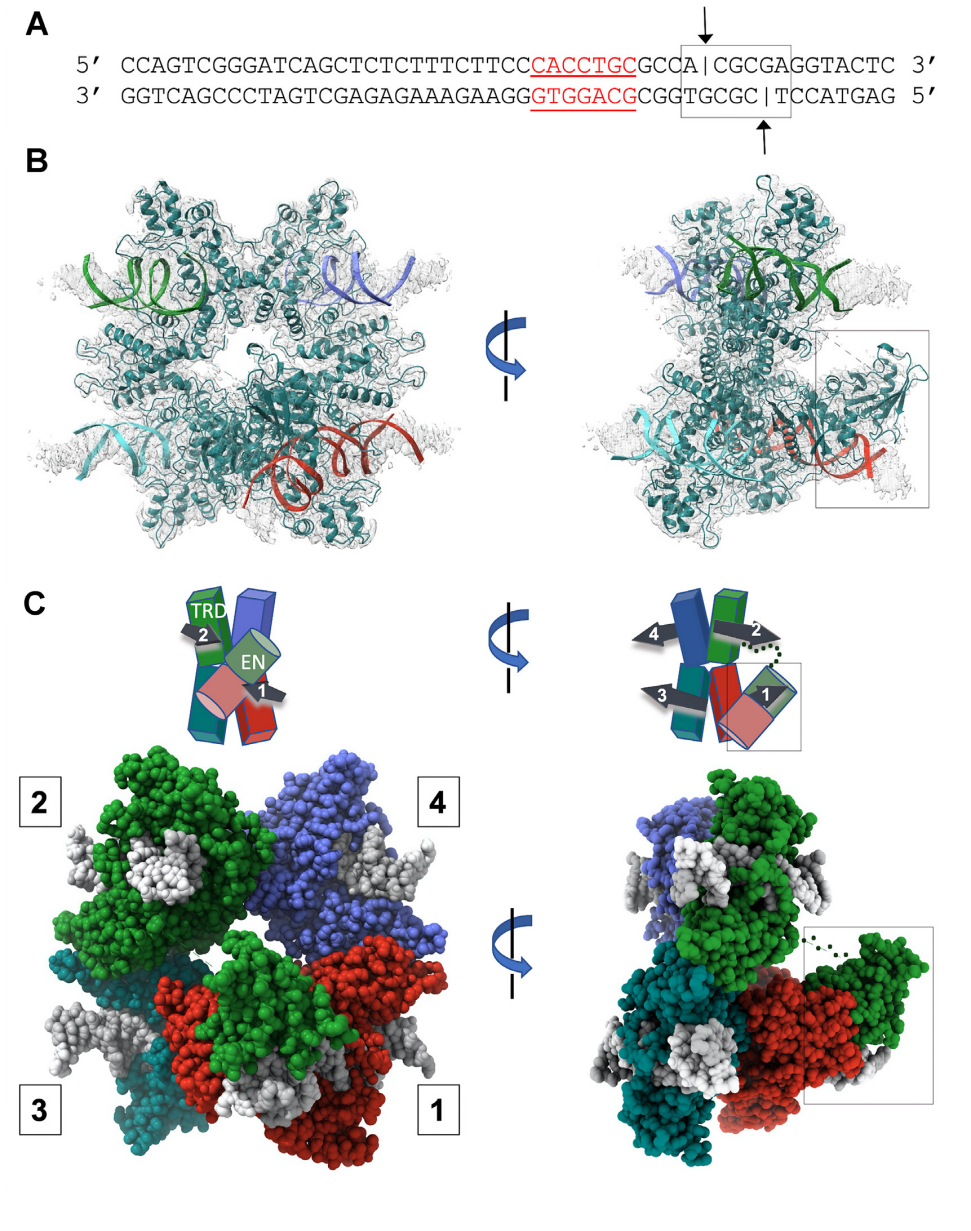
CryoEM analysis of DNA-bound PaqCI. (**A**) Sequence and basepair arrangement of DNA duplexes used to form a DNA-bound enzyme complex. The duplex consists of 50 complementary basepairs and includes both the enzyme's 7 bp target site (red underlined bases) and its downstream cleavage sites on the top and bottom strands (black lines and arrows, 4 and 8 bp downstream from the final basepair of the target site). (**B**) CryoEM electron density corresponding to the DNA-bound PaqCI enzyme. Each of the four double-stranded DNA oligoduplex is independently colored to match that of its bound monomer shown in Panel C. The protein tetramer is colored in teal. The right image is rotated 90° around the y-axis and the endonuclease (EN) domains engaged for cleavage are boxed. (**C**) Cartoon representation and space filling model of DNA-bound enzyme structure and domain organization. The target recognition domains (TRDs) are represented by rectangles and the EN domains are shown as cylinders. DNA duplexes and their directionality (5’ to 3’) are denoted with black arrows in the cartoon. Each TRD is engaged with one DNA duplex, via contacts to bases and neighboring backbone atoms distributed across the target site. The downstream region of each bound DNA, including the sites of cleavage, extend away from the TRD tetramer. The DNA duplexes engaged to each protein dimer (DNA 1 and 2 on one side of the complex, and DNA 3 and 4 on the opposite side of the complex) are roughly parallel to one another, as indicated in the cartoon schematics. All four ENs have been released from their positions in the DNA-free apo-enzyme structure. Two ENs (from TRDs colored blue and teal bound to DNA 3 and 4) are disordered and are unobservable in the density map. The other two ENs (from TRDs colored red and green, *cis* and *trans*, bound to DNA 1 and 2) are observed to have undergone significant motions resulting in their engagement around the cleavage site on one bound DNA duplex. The two EN domains engaged for cleavage are boxed. Size exclusion chromatography traces of DNA-bound complexes, representative negative stain EM class averaged images, and electron density surrounding the DNA cleavage site complexes are further illustrated in Supplementary Figure S3.

A 50 bp double-stranded DNA construct (Figure 4A) containing the enzyme's target site and sufficient downstream duplex to extend past the enzyme's cleavage site was used to generate a DNA-bound enzyme complex in the presence of calcium (which facilitates the formation of a productive cleavage complex but inhibits cleavage). The formation of the bound complex was validated via SEC analyses of the protein–DNA mixture (Supplementary Figure S3a) and subsequent negative stain and CryoEM images, which clearly indicated the presence of multiple bound DNA molecules extending from individual enzyme particles (Supplementary Figure S3b).

**Figure 5.**
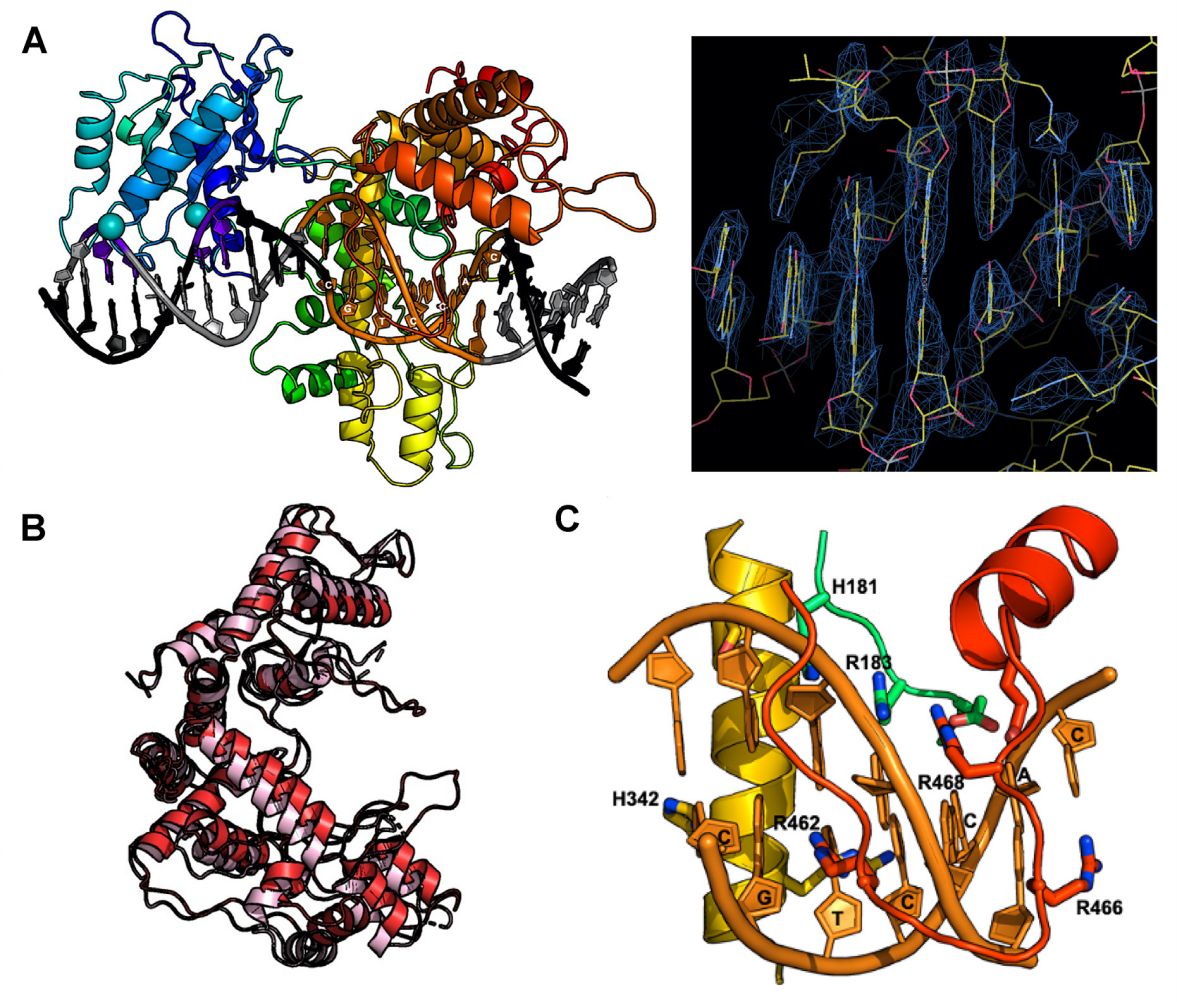
Target site binding by PaqCI. (**A**) Cartoon representation of *cis* DNA-bound protein subunit and CryoEM electron density across the DNA target site bases. The protein ribbon is colored in a spectrum, with the N-terminal endonuclease (EN) domain in shades of blue, and the C-terminal target recognition domain (TRD) colored in shades of yellow, orange and red. Two calcium ions are shown as spheres. (**B**) Superposition of DNA-free and DNA-bound TRDs (pink and red, respectively) show a slight domain closure and ordering of several DNA-contacting loops. (**C**) A close up of one of the DNA-contacting loops of the TRD, displaying the base specific read out of the enzyme using primarily arginine residues.

A CryoEM density map of the DNA-bound enzyme (Figure 4B), corresponding to approximately 3.0–3.6 Å resolution, was generated from 1,291 micrographs and over 800 000 particles. The real space density was well-resolved throughout the DNA-bound tetramer, including the DNA target site (Figure 5A) and the downstream cleavage site (Supplementary Figure S3c). The lowest resolution of the CryoEM map corresponds to the distal ends of the DNA molecules and the linker spaces between the EN and TRD domains (Supplementary Figure S4b).

3D variability analysis of the CryoEM DNA-bound tetramer map shows a reduction in overall conformational sampling. The largest motions observed within the particles correspond to the regions of the bound DNA molecules that extend away from their binding sites and the protein assemblage (Supplementary Movie S2). The tetramer appears to remain in a relatively rigid conformation in solution once it is bound to DNA with minimal ‘breathing’ of the core tetrameric assembly as compared to its unbound counterpart.

***Target binding***. All four TRDs within the PaqCI tetramer are individually engaged with four corresponding double-stranded DNA duplexes (Figure 4B) through contacts with the enzyme's target site sequence to form a complex to cleave a single DNA duplex. The binding of DNA imparts minimal rearrangement of the TRD tetrameric assembly (Supplementary Figure S5a). Within each TRD dimer within the larger ‘dimer of dimers’ tetrameric assemblage, two bound DNA molecules are oriented in a parallel arrangement relative to one another, as indicated by the numbered arrows in the cartoon diagram above the space filled structures of Figure 4C. The opposing pair of bound DNA duplexes are also oriented in a parallel arrangement relative to one another. The two pairs of bound DNA duplexes (labeled 1 and 2 versus 3 and 4 in Figure 4C) point in opposite directions from one another.

The TRD in each enzyme monomer in the PaqCI tetramer displays small, localized conformational changes as it wraps around the target site (Figure 5B and Supplemental Figure S5a). The conformational changes within the TRDs upon double-stranded DNA binding correspond to an overall rmsd between unbound and bound states of approximately 2 Å, with the largest movement corresponding to a slight closure of α-helices and corresponding ordering of adjacent DNA-contacting loops that are unobservable in the DNA-free PaqCI enzyme (Figure 5B). Multiple residues in each TRD are engaged with individual bases and backbone atoms within each target site, forming a site-specific recognition complex (Supplemental Figure S5b). In particular, one of the three DNA-contacting loops contains four arginine residues, each of which interacts with the backbone and/or bases of the target site in a sequence-specific manner (Figure 5C). Complementing these interactions, an adjacent α-helix inserts into the major groove near the 7 bp recognition site. This helix is interrupted by a loop of 14 amino acids that is also used for DNA recognition. Seventeen residues are indicated by DNAproDB (45) to be directly involved in recognition of the target site (either via backbone or residue contacts). The DNA contacts exhibited by each subunit are similar, regardless of whether the corresponding TRD is associated with a visible DNA-bound endonuclease (Supplementary Figure S5b).

**Figure 6.**
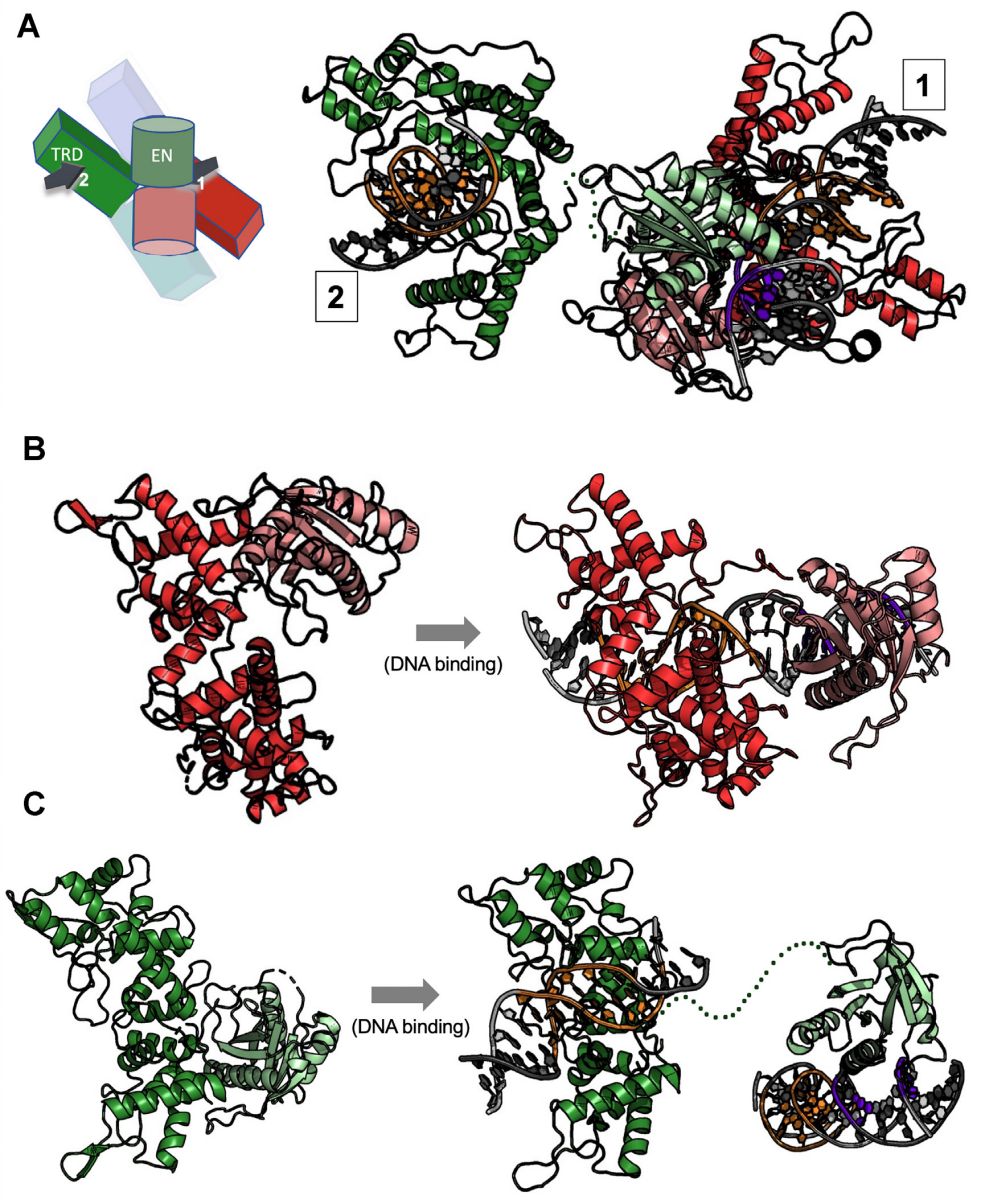
Endonuclease motions and engagement on DNA. (**A**) Ribbon diagram and cartoon schematic of *cis* and *trans* protein subunits (as shown in Figure 4C) that position their respective endonuclease (EN) domains on both strands and cleavage positions on a single bound DNA duplex. In the cartoon schematic the DNA duplexes are drawn as arrows and numbered according to their protein subunit (*cis* = 1, *trans* = 2) (as shown in Figure 4C). EN domains are colored in a lighter shade than the target recognition domains (TRDs). The cleavage sites of the DNA duplex are shown in purple and the target site in orange. The 16 amino acid linker between the *trans-*acting EN domain and its C-terminal TRD is poorly ordered in the model and are represented in the figure as a dotted green line. (**B**) EN domain motion of the *cis*-acting endonuclease domain (red subunit in Figure 4C) acting on DNA 1. The EN domain (pink) swings about 180° about the axis to align with the cleavage site of DNA 1 to cut four bases from the target site while the TRD (red) stays fixed except for slight closure of the helices to bind DNA. ***Panel c***: EN domain motion of the *trans*-acting endonuclease (green subunit in Figure 4C) acting on DNA 1. The TRD (green) is bound to a separate DNA duplex (DNA 2) than the one bound by the *cis-*acting EN and the *trans*-acting EN engage for cleavage (DNA 1). The EN domain (light green) reaches across the belt of TRDs to locate the cleavage site of the DNA 1 to cleave.

**Figure 7.**
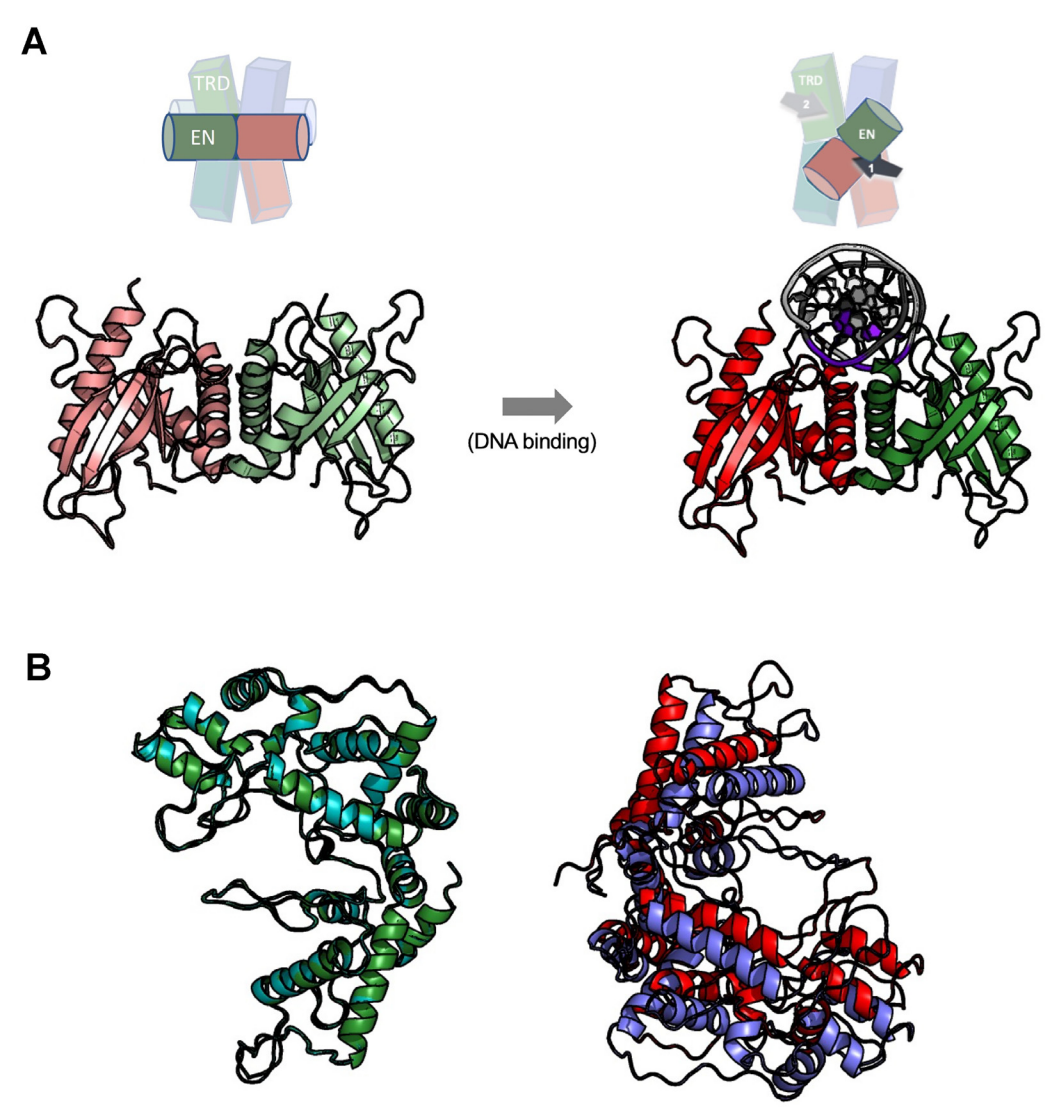
Contacts between EN domains in the enzyme tetramer in the presence and absence of a DNA cleavage site are conserved. (**A**) Endonuclease (EN) domain dimer organization in the DNA-free and DNA-bound PaqCI structures (left and right, respectively) shown as cartoon and ribbon diagrams. The dissociation of the two domains from their respective target recognition domains and subsequent re-association with the two DNA strands at the sites of cleavage result in nearly identical contacts and association between the two domains (backbone rmsd 0.8 Å). (**B**) Superposition of the ‘cleaving’ and ‘non-cleaving’ DNA-bound target recognition domains (colors correspond to Figure 4C) indicates that an asymmetry in the TRD tetrameric structure is induced when the *cis-* and *trans*-acting endonuclease domains associate with a DNA cleavage site associated with one of the two enzyme dimers.

***A single pre-cleavage complex is formed around one bound DNA duplex***. In the DNA-bound complex, all four endonuclease domains (ENs) have dissociated from their original positions, where they were originally sequestered against their corresponding target recognition domains (TRDs). Two of the ENs, extending from one of the two enzyme dimers in the protein tetramer, have reformed a catalytic EN dimer around the cleavage site of one of the bound DNA duplexes (indicated by boxes in all panels of Figure 4 and in Figure 6). Although both EN domains each move significantly to position themselves around their newly acquired cleavage site, the DNA-bound ENs are re-dimerized in a manner very similar to their association in the DNA-free apo-enzyme, with their protein–protein interactions largely re-established via polar contacts between α-helices of the two EN domains. The backbone rmsd between the EN domain dimer pair in the unbound and DNA-bound structures is approximately 0.8 Å (Figure 7A).

One of the two DNA-bound EN domains (colored red in Figures 4C, 6, and 7) is engaged with the DNA cleavage site in *cis* (i.e. that domain extends from the TRD that is engaged with the same DNA duplex). The other EN domain (colored green) is engaged with the opposing strand of the same cleavage site in *trans* (i.e. that domain extends across the dimer interface from a TRD bound to a different DNA duplex and target site). The remainder of this manuscript refers to the two DNA-bound EN domains as the *cis*-acting and *trans*-acting EN domains.

The *cis*-acting EN domain is positioned appropriately for cleavage of the phosphodiester located 4 bp from the target site, while the *trans*-acting EN domain (which has moved a much longer distance to re-establish contact with its partner) is positioned appropriately to cleave the phosphodiester bond 8 bp from the target site on the opposing strand (Figure 6A). Whereas the *cis* monomer twists around the duplex by about 180° to orient the EN domain over the phosphate of interest (Figure 6B and Supplementary Movie S3), the *trans* monomer reaches approximately 40 Å across the dimer interface, thereby reforming a new EN dimer and positioning itself appropriately for cleavage of its target phosphate (Figure 6C and Supplementary Movie S4). The *trans* monomer relies on a linker of 16 amino acids that connects it to its own DNA-bound TRD to reach its cleavage site and appears to be near the maximum motion away from its TRD that would be sterically permitted by its linker.

In the complex between the DNA target site and its re-dimerized *cis*-acting and *trans*-acting ENs, each active site is positioned appropriately around the precise phosphate groups corresponding to the known cleavage pattern for the PaqCI enzyme (Figures 6 and 7A). The EN domains each coordinate a metal ion in direct contact with the phosphate to be cleaved (Supplementary Figure S3c). Three conserved catalytic residues (D54, E73 and K75) complete a canonical endonuclease active site via coordination of bound metal ions and appropriate positioning for proton transfer and stabilization of the phosphoanion transition state of the hydrolysis reaction.

***The opposing EN domains are disordered***. The opposing two EN domains (also previously packed against their own TRDs in the DNA-free apo-enzyme), which are also released from their TRDs as a result of DNA binding, are unobservable and appear to be disordered. Even though all four DNA duplexes and their corresponding TRDs are well resolved, no class exists within the 2D classifications that display more than the two DNA-bound EN domains described above. If the unobservable EN domains are able to simultaneously associate with their own DNA target sites, that interaction would appear to be too short-lived in the trapped pre-cleavage state described here for the CryoEM analysis to visualize.

The asymmetry between the two sides of the DNA-bound tetramer (leading to the formation of a cleavage-ready complex to a single-bound DNA on only one side of the enzyme tetramer) may imply that engagement of EN domains on one bound double-stranded DNA imposes a subtle asymmetry between the two enzyme dimers that leads to a corresponding inability of the opposing EN domains to reach far enough to easily form a simultaneous, comparable interaction with a DNA cleavage site on the opposite side of the enzyme assemblage. This asymmetry is evident when comparing the relative conformation of the ‘DNA-cleaving’ TRD dimer with the opposing TRD dimer. The two dimers in the DNA-bound structure display different subunit packing, corresponding to a backbone rmsd of approximately 3 Å between the unaligned TRDs in a superposition (Figure 7B). Furthermore, the superposition of individual TRDs from opposing sides of the assemblage demonstrates that the conformational change induced by DNA binding and EN engagement involves a rigid body motion of the two TRDs within a dimer relative to one another.

These observations appear to also correlate with correlated motion analyses of the CryoEM maps for the unbound enzyme tetramer (Supplementary Movie S1), which appears to indicate an oscillation across the dimer-of-dimers interface that corresponds to sampling of transient asymmetry between the two sides of the enzyme assemblage. The asymmetry between the two sides of the enzyme suggests that the enzyme cleaves in a sequential manner and is unable to be positioned to cleave two sites simultaneously.

The atomic interactions between the monomers of the DNA-free and DNA-bound tetrameric structures were analyzed using the PISA software to provide additional details of the overall interactions required for enzyme tetramerization and DNA cleavage (46). While the tetramer is bound to DNA, the only contacts holding the quaternary structure together are in the TRDs, preserving the TRD ‘ring’ in both structures (Supplemental Figure S5a). In the unbound structure, the EN domains are held against the TRD ring via contacts between periphery EN loops and a single TRD loop on its neighboring and adjacent monomer. This locks the catalytic domain against the interior of the tetramer and prevents it from cleaving nonspecific DNA duplexes. The EN domains of the DNA-bound structures make no contact with the TRDs, but the contacts between EN domains are preserved (Figure 7A).

### Further biochemical experiments examining cleavage mechanism

**Figure 8.**
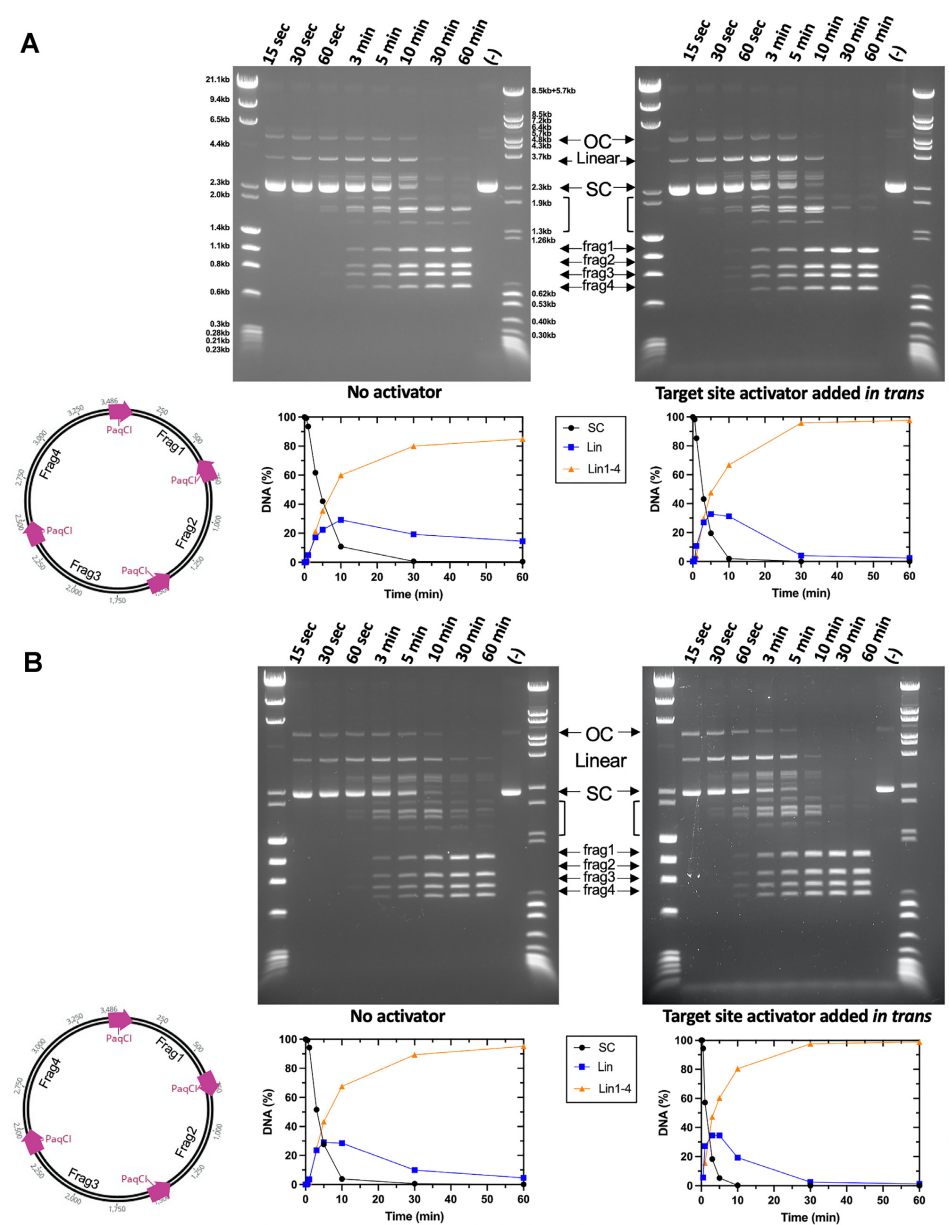
Time-dependent digestion of plasmids with four PaqCI target sites indicate a sequential cleavage at individual sites. (**A**) Cleavage of a four-site substrate with and without an activator added in *trans*. The no activator reaction (left gel) contained 37 nM DNA target sites and 17.2 nM of monomer PaqCI per 37nM DNA target sites incubated in 50 μl buffer. The activator reaction (right gel) had the same substrate and PaqCI enzyme concentrations with 80 nM of added activating oligoduplex incubated in 50 μl buffer. Digest time points were quenched at 0.25, 0.5, 1, 3, 5, 10, 30 and 60 min. The mobilities of the super coil (SC), single-cut linear (Linear), intermediates (brackets), and the complete-cleavage linear (frag1, frag2, frag3 and frag4) forms of the plasmid are indicated in the middle key with arrows corresponding to their related bands. Intermediate bands correspond to a less than fully cleaved plasmid; either 1, 2 or 3 cleavage events at any of the sites available. To the left of the gels is a cartoon of the substrate and reaction products shown in the gel. The intensities of each substrate and product band in the gels were quantitated using ImageJ (54) software and calculated as rough percentages to be shown graphically. The key in the middle shows supercoiled DNA substrate (purple circles, SC), linearized DNA (blue square, Lin), and final product linearized DNA (orange triangle, frag1–4). Two molecular weight ladders were used in this experiment: (i) Lambda-HindIII/PhiX174_HaeIII which spans 0.2–23.1 kb, and (ii) Lambda-BstEII/pBR322-MspII which spans 0.3–8.5 kb. This experiment was performed once. (**B**) The cleavage of an alternative four-site substrate with and without an activator added in *trans*. Reactions were conducted as in Panel a. This experiment was performed once.

**Figure 9.**
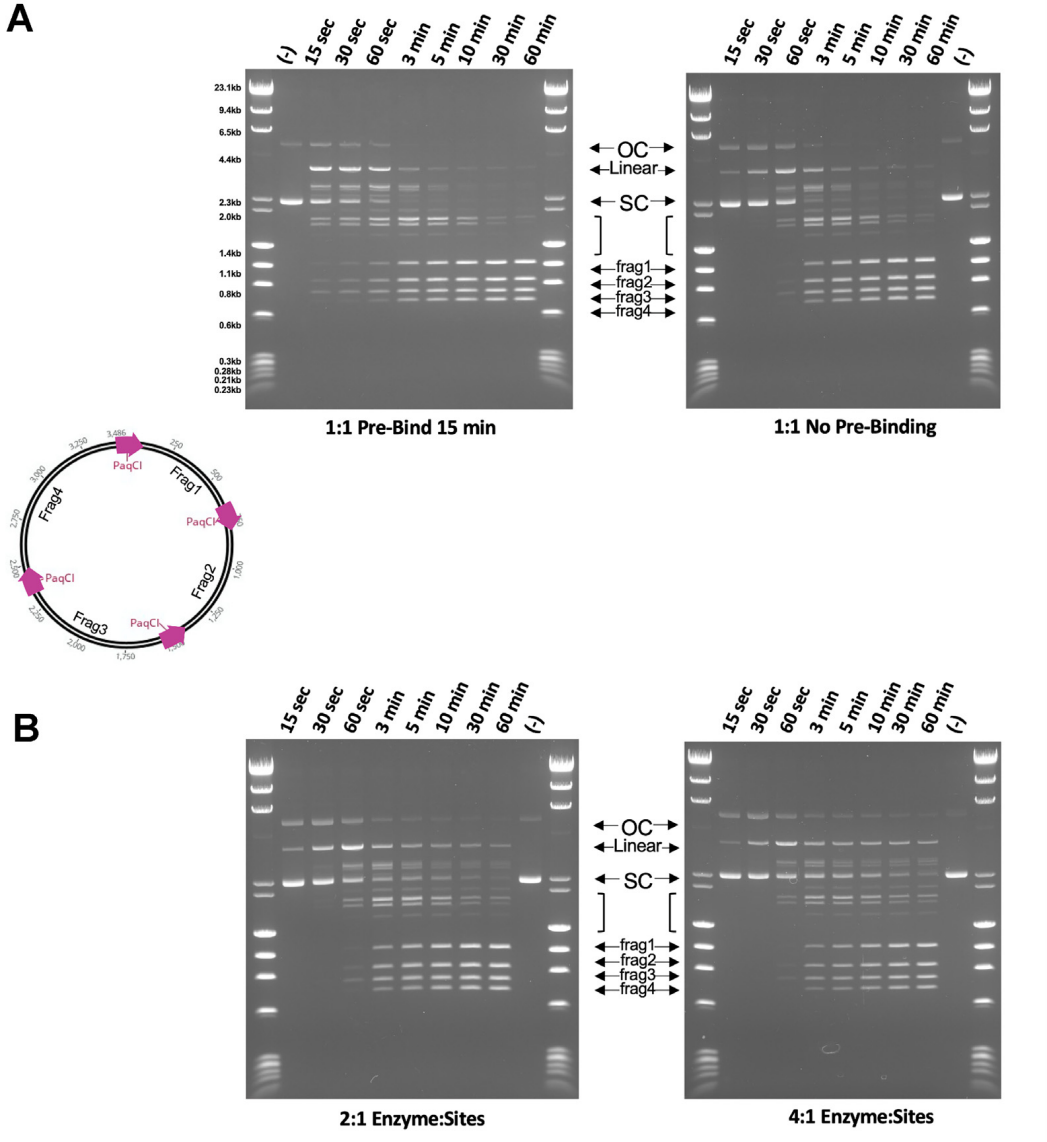
DNA pre-bound at all four sites in the PaqCI tetramer is cut sequentially. (**A**) Cleavage of a four-site plasmid substrate either pre-bound (left) or not pre-bound (right) with a 1:1 enzyme-binding-site to DNA-sites ratio. The pre-bound reaction (left gel) contained 37 nM DNA target sites and 37 nM of PaqCI per 37 nM DNA target sites in 50 μl buffer lacking Mg^2+^ ions. The enzyme was allowed to bind for 15 min at 37°C. An aliquot was removed (time 0) and the cleavage reaction was initiated by adding Mg^2+^ to 10 mM. The no pre-binding reaction (right gel) had the same substrate and PaqCI enzyme concentrations in normal buffer, with time points started upon enzyme addition. Digest time points were quenched at 0.25, 0.5, 1, 3, 5, 10, 30, 60 min. The mobilities of the super coil (SC), single-cut linear (Linear), intermediates (brackets), and the completely-cleaved linear (frag1, frag2, frag3 and frag4) forms of the plasmid are indicated in the middle key with arrows corresponding to their related bands. Intermediate bands correspond to a less than fully cleaved plasmid; single, dual, triple and complete cleavage events. To the left of the gels is a cartoon of the substrate and reaction products shown in the gel. Molecular weight ladders were Lambda-HindIII/PhiX174_HaeIII which spans 0.2–23.1 kb. These experiments were performed once. (**B**) Same reaction conditions and plasmid, as in Panel a right side, except that the PaqCI enzyme concentration was increased to 2:1 (74 nM enzyme to 37 nM DNA sites) or 4:1 (148 nM enzyme to 37 nM DNA sites) ratio of enzyme to DNA sites. These experiments were performed once.

The structural analyses described imply that PaqCI may display a random, sequential mechanism in which one double-stranded DNA at a time is cleaved within a fully-formed reaction synapse containing multiple bound DNA target sites. The cleavage activity of PaqCI against DNA containing multiple target sites was further probed in a final experiment using plasmid substrates harboring four target sites in two different relative arrangements (illustrated in the schematics in Figures 8 and 9). Digest time points were quenched at varying time points from 15 s to 1 h under the same conditions as the experiments performed in Figure 2. Each target site is placed between 700 and 900 bp from each other, allowing for sufficient flexibility of the plasmid DNA relative to the known persistence length of DNA (47).

In the cleavage experiments using substrates containing four target sites (Figures 8 and 9), the super-coiled (SC) species rapidly decreases as the initial single-cut linearized product and dual- or triple-cut intermediate products appear, followed by appearance of the final product fragments (frag1–4) corresponding to complete digestion at all four sites. As observed for the two-site substrate in Figure 2, there does not seem to be a major difference in the performance of the enzyme when cleaving plasmids with different arrangements of the four target sites, in the absence of the activator. Prior to the formation of the complete digestion species (frag1-4), there is an equal accumulation of bands (linear) that reflect the intermediate states of cleavage. Each band larger than the final products (frag1–4) represents a different combination of cleavage events across the four-site plasmid, associated with single-, dual-, and triple-cut substrate generated prior to the complete cleavage at all four sites. In the activator experiments, seen in the right-most gels in the figure, cleavage is remarkably similar, with a slight increase in the linear plasmid species resulting from a single cleavage event as the reaction proceeds, and more complete cutting as the reaction proceeds.

To further investigate the sequence of cleavage events, the four-site substrate was pre-incubated at a 1:1 enzyme TRD to DNA target site ratio in the absence of Mg^2+^ ions to allow binding, preferably with one tetramer binding all four sites in one plasmid. Cleavage was then initiated by adding Mg^2+^ and time points taken (Figure 9A). Cleavage occurred more quickly following pre-binding the enzyme but produced the same fragments resulting from single, dual, triple, and complete cleavage events, indicating sequential rather than coordinated cleavage events even when all four target sites are bound in the same enzyme tetramer. The substrate is cut nearly to completion, though a small amount of partially cut substrate remains. Increasing the ratio of enzyme TRDs to sites above 1:1 resulted in an increase in the partial cut fragments (less complete cutting), with the higher 4:1 enzyme-TRD-to-sites condition having more partial cutting than 2:1 (Figure 9B).

## DISCUSSION

To bias cleavage towards foreign invading DNA, many restriction endonucleases (REases), including some from the Type II class, are thought to require multiple unmodified DNA targets to be brought together into a cooperative reaction ‘synapse’ before nuclease activity is licensed (48–50). The requirement of a reaction synapse with multiple bound targets and a mechanism requiring one or more *trans*-acting endonuclease (EN) domains within that synapse is a particularly common feature of many bipartite REases with separate EN domains and target recognition domains (TRD) (48,49,51,52). However, such enzymes (spanning both the Type IIS and Type IIG REase subfamilies) span a vast array of architectures and mechanisms. Previous structural and functional studies on such enzymes, such as FokI and DrdV, revealed features that are unique to each enzyme while still cleaving at their targets with high specificity and fidelity. Despite this wealth of research, a high-resolution view of the cleavage synapse formed by a canonical Type IIS enzyme has yet to be described. To address this point, the activity and structures of the Type IIS enzyme PaqCI in the presence and absence of bound DNA were determined.

### Mechanistic implications

Size Exclusion Chromatography (SEC) and CryoEM analyses independently indicate that DNA-free PaqCI exists as a preformed tetramer, which is tasked with scanning incoming foreign DNA for the enzyme's 7 bp asymmetric target site. Like most DNA binding proteins, including REases, the enzyme multimer is assumed to find its target via a search mechanism involving subcellular localization near the host and/or foreign DNA and corresponding rapid association and dissociation along the DNA helix combined with limited episodes of one-dimensional (1D) diffusion (‘sliding’) (6). However, in contrast to ‘simpler’ REases that have been proposed to conduct their target searches as monomers (and then to self-associate upon encountering individual target site, thereby forming a higher-order DNA-bound assemblage), PaqCI appears likely to sequentially load individually encountered target sites into the preformed enzyme multimer. How the kinetic and dynamic details of such target search mechanisms differ from one another might be an interesting future topic for detailed examination.

The PaqCI-DNA complex is a tetramer composed of a ‘dimer-of-dimers’, with each pair of subunits on opposing sides of the assemblage independently placing their pairs of bound DNA targets and corresponding cleavage sites into a parallel arrangement. The dimer-of-dimers, in the DNA-bound complex trapped in a pre-cleavage state is comprised of a “cleaving dimer” (with its two EN domains associated with a single cleavage site in *cis* and *trans*) and a “non-cleaving dimer” (with its two EN domains apparently disordered). This result implies that the DNA-bound complex might be sterically unable to simultaneously form two cleavage-competent reaction complexes, perhaps due to limitations in the extension and ‘reach’ of the *trans*-acting EN domain. The CryoEM structure of the DNA-bound complex indicates that the engagement of a cleavage site on one side of the complex by two EN domains appears to generate an asymmetry in the enzyme-DNA complex that might prevent the corresponding engagement of EN domains around an opposing DNA cleavage site. Correlated motion analyses of the DNA-free enzyme tetramer appear to agree with this speculation: in that analysis, the enzyme seems to display a dynamic equilibrium between two slightly asymmetric conformations that alternately tilt towards one or the other of the two enzyme dimers in the enzyme tetramer (Supplementary Movie S1).

**Figure 10.**
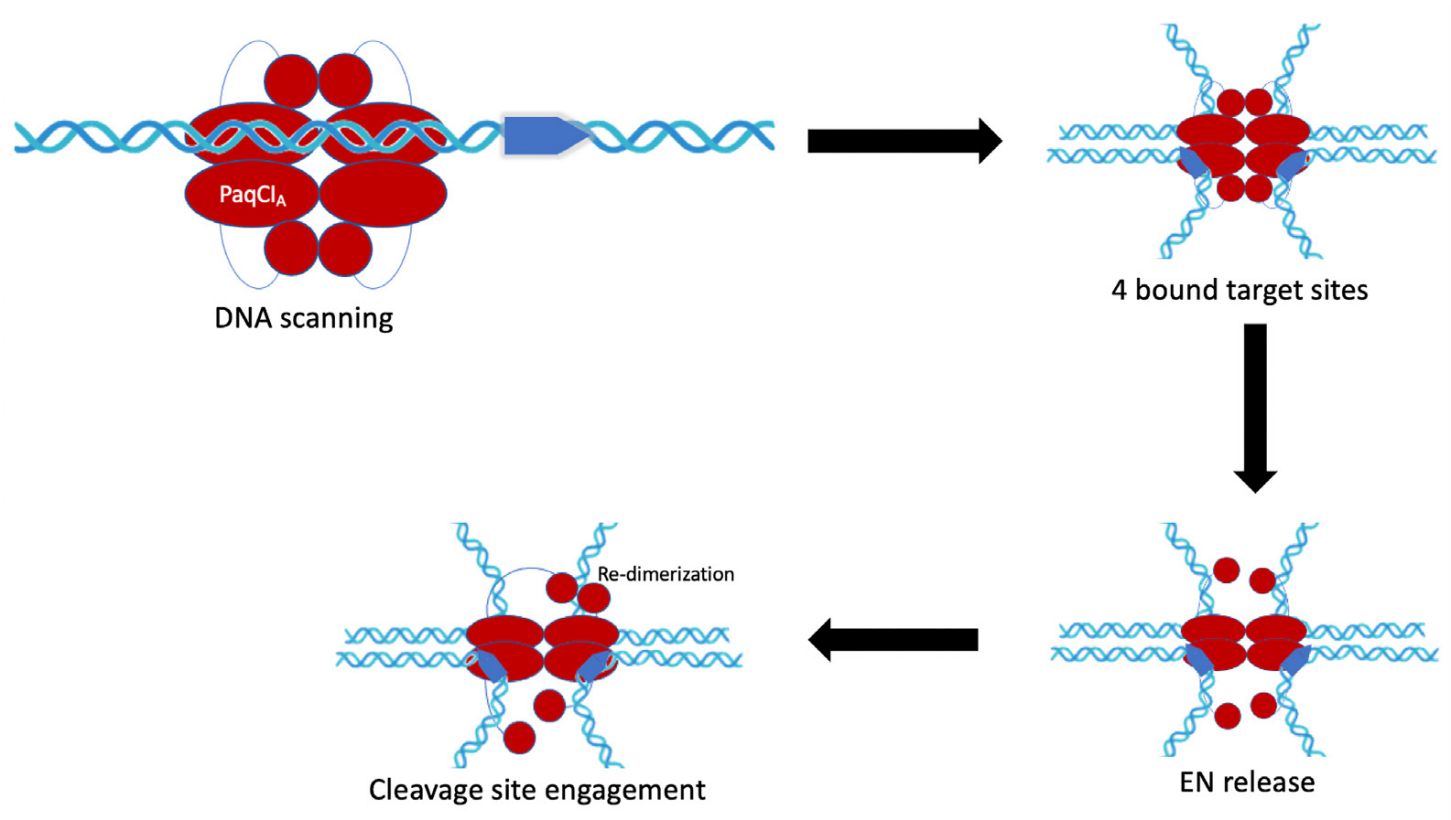
Schematic of PaqCI’s mechanism as suggested by kinetic and structural data. The PaqCI tetramer is shown in the first panel in red and a double-stranded DNA in blue with a single target site. The point of the target site reflects the direction of the asymmetric target sequence. Each monomer (i.e. PaqCI_A_) of the oligomer is represented by an oval (TRD), a circle (EN domain), and a thin line connecting them (linker). PaqCI operates by scanning the DNA for four identical asymmetric target sites that are 7 bp long. Once it finds four identical target sites, it brings them together in a synapse using the TRDs. The binding of the four target sites triggers the release of all the EN domains from the TRDs. As the EN domains are free in solution, two find the cleavage site on one double-stranded DNA and engage that site for cleavage.

This observation that only one cleavage competent dimer of two EN domains positioned at one cleavage site is formed in the DNA-bound pre-cleavage complex leads to a prediction that the enzyme may follow a sequential cleavage mechanism (illustrated schematically in Figure 10), wherein each DNA within the complex of four bound target sites would be cleaved in turn (although presumably in random order relative to the first cleavage event). *In vitro* kinetic studies performed on a dual-site plasmid (Figure 2) agree with the hypothesis generated from the structural studies. In both the head-to-tail and head-to-head experiments, the supercoiled (SC) plasmid is swiftly cleaved once to form the full-length linear (Lin) form. The Lin form appears at a faster rate than the large and small linear (Lin1 and Lin2) forms, implying that one site is cleaved prior to cleavage of a second site. When testing the enzyme against two different four-site plasmids (Figures 8 and 9) a single-cut full length linear plasmid band is generated first as the uncut supercoiled plasmid is cleaved, followed by all the linear bands (which represent any combination of less than complete four-site cleavage) that appear at the same relative rate, suggesting that PaqCI does not prefer a particular site to cleave first and that it cleaves only one site at a time. Even with the availability of four sites in each substrate plasmid and pre-incubation with no Mg^2+^ to allow a tetramer to bind a site to each of its four TRDs, cleavage produces the same fragment pattern, without any nicked species, that indicates sequential cutting of one site at a time.

The structure explains the very partial cutting observed on a single-site DNA substrate, where binding to a single DNA site would not activate two opposing endonuclease domains to enable cutting. At minimum an enzyme tetramer would need to bind two single-site DNAs, and the second DNA would have to be bound in the correct opposing enzyme monomer (a one in three chance), to activate two endonuclease domains for cleavage. Complete cutting of a single site substrate is observed in the presence of excess *in trans* recognition site oligo because when the enzyme binds the single site substrate DNA the other three binding sites in the tetramer can be occupied by the *in trans* activator oligo to license the release of the endonuclease domain opposite the substrate-bound monomer to form the required catalytic dimer to cut the single site substrate.

The observation that roughly 1:1 enzyme binding site to DNA target site is required for complete cleavage suggests there is little enzyme turnover. The DNA binding site remains intact after cleavage, so if the enzyme has relatively long-lived DNA binding persistence this could explain a lack of turnover. The observation that the small in trans oligo optimally stimulates cutting at roughly a 5:1 ratio of oligo to enzyme binding sites suggests the small oligo may bind much less tightly and have higher turnover than normal long DNA molecules.

The same stereochemical constraints that appear to prevent simultaneous engagement of multiple cleavage sites in the enzyme–DNA assemblage might also dictate the cleavage position of PaqCI (which cleaves the top and bottom strands 4 and 8 bp downstream from its bound target site, respectively). DNA cleavage clearly requires the re-dimerization of the EN domains around the cleavage site, which in turn dictates the 4 bp separation between individual strand nicks. Modeling the possible engagement of the EN dimer either closer to, or farther away from the bound target site indicates that (i) the *cis-*acting monomer cannot easily engage DNA closer to the bound target site without imposing a significant clash with its own TRD, and (ii) the *trans*-acting monomer appears to be extended to near the limit of its potential reach across the enzyme tetramer. The combination of these two stereochemical constraints would thereby limit the association of the two EN domains to the precise phosphate groups corresponding to the enzyme's cleavage pattern.

The closely related isoschizomer BspMI (which shares 39% amino acid identity to PaqCI) behaves quite similarly: it exists as a preformed tetramer before binding its target DNA, maintains that same quaternary assemblage after binding DNA, and cleaves each bound DNA duplex in both strands without intermediates cut at a single strand of the DNA duplex (48,52,53). Kinetics of BspMI also demonstrate an increase in the enzyme's cleavage efficiency when a DNA duplex containing the enzyme's recognition site is added as a *trans*-activator (48,52). Additionally, a single turn-over event requires two independent double-stranded cleavage events, suggesting that within a tetrameric complex harboring a dimer-of-dimer architecture, one enzyme dimer at a time cleaves its bound DNA duplexes while the other waits for its turn, just as seen with PaqCI. This could mean that PaqCI also only requires one dimer of the dimer-of-dimers to be bound at one time to cleave the DNA duplex, and the structure shown here with all four sites bound is a product of DNA saturation in the experimental preparation. However, in kinetic experiments, BspMI was reported to cleave both DNA duplex sites rapidly, with only a small accumulation of a single-cut linear species prior to cleavage at the second site (48,52). Acc361, another isoschizomer, produces an accumulation of a single-cut linear species, similar to PaqCI, cleaving the dual site plasmid with two kinetically separate cleavage events, furthering the knowledge that even amongst Type IIS isoschizomers there is a high degree of variation (13,18,48,52). Additionally, BspMI, as well as FokI, exhibit some preference to the orientation of the DNA sites (head-to-head or head-to-tail). Our experiments with PaqCI suggest that there may be a slight preference for head to tail sites at the 700bp separation tested, but more kinetic analyses are necessary to definitively address this point.

The recently visualized Type IIG enzyme DrdV displays similarity to PaqCI but with significant differences in many of the underlying details of its action. DrdV also contains an N-terminal EN domain and a C-terminal TRD, recognizes and binds an asymmetric site (5’ CATGNAC 3’), cleaves downstream of its target, does so as a tetrameric enzyme assemblage engaged with multiple bound DNA targets, and relies on the combined action of *cis*- and *trans*-acting enzyme domains to generate individual double-strand breaks. However, unlike PaqCI (or FokI, described below) DrdV generates a two-base 3′ overhang (cleaving top and bottom strands 10 and 8 bases downstream of the target, respectively). Also, unlike PaqCI and FokI, it incorporates its cognate methyltransferase domain and activity into the same protein chain (located between the EN and TRD domains) and then establishes a kinetic competition between slow methylation activity at individual bound target sites relative to rapid cleavage of a multi-target enzyme assemblage, to bias the two competing outcomes towards self- or nonself-DNA. Finally, DrdV exists as a monomer in solution prior to DNA target binding and couples the formation of a cleavage-competent tetrameric assembly to DNA target acquisition and binding. Unlike both FokI and PaqCI, three separate DrdV subunits are engaged on a single bound DNA duplex and required for cleavage: a target-bound TRD domain from one subunit, plus two separate *trans*-acting EN domains that are assembled at the cleavage site downstream of that target. Despite those variations, DrdV still fundamentally requires *cis/trans* collaboration to enforce the requirement for multiple bound target sites for cleavage.

FokI is the most well-studied Type IIS REase to date (23–26). While also acting as a canonical Type IIS REase, it displays significant differences as compared to PaqCI (Supplemental Figure S6a). Its domain organization is reversed; it exists in solution primarily as a monomer in the absence of bound DNA, it recognizes a shorter five-base asymmetric target site, and it cleaves top and bottom strands farther downstream (9 and 13 bases, respectively) from its bound target site than does PaqCI (which cleaves the top and bottom strands only 4 and 8 bases away from the bound target). Whereas PaqCI accomplishes this within the context of a larger, preformed tetrameric architecture, FokI appears to instead generate a similar cleavage synapse via the transient formation of a DNA-bound dimer. Prior studies of FokI, therefore, provide an important point of comparison to understand the Type IIS REase family.

While these studies of FokI have not generated an atomic-resolution structure of a cleavage-competent enzyme assemblage, a well-validated model (Supplemental Figure S6b; left) of its cleavage complex, corresponding to a transient DNA-bound enzyme dimer, has been proposed based on a variety of biophysical and structural analyses (26–32). This model indicates that two independent enzyme monomers are bound to two separate DNA target sites via their individual TRDs. Their corresponding EN domains are wrapped around one of the DNA duplexes (thereby acting jointly in *cis* and *trans*, as observed in the structure of PaqCI). However, in that model, the FokI TRDs are not engaged in a higher-order complex with one another, and the only contact between the protein subunits involved the DNA-associated EN domains.

While the general features of how they cleave DNA (by placing *cis*-acting and *trans*-acting endonuclease domains on one bound DNA duplex at a time) are similar, the underlying details of how they sequester their EN domains in the absence of foreign DNA and the structural rearrangements that each dimer must undergo to enter similar cleavage-competent DNA-bound complexes appear to differ significantly. FokI and PaqCI share a similar EN domain dimer organization and place them in similar orientations around their cleavage sites, such that both enzymes generate four-base 5′ overhangs (Supplemental Figure S6a, b). In both cases, the EN dimers in the DNA-free and DNA-bound states share strong structural similarity (corresponding to ∼0.2 Å backbone RMSD between pre- and post-DNA binding states for PaqCI; Figure 7A). This EN domain dimerization motif is common amongst other Type II enzymes, such as BamHI, and is notable considering the significant differences in topology between these proteins (24). To do so, PaqCI and FokI each rely on a flexible 16-residue linker between the EN and TRD domains. This peptide region allows the *trans-*acting PaqCI EN domain to extend almost 40 Å across the tetramer interface to re-engage with its *cis*-acting EN partner at the DNA cleavage site. In contrast, FokI does not appear to require the full length of its linker to contact its cleavage site, only needing to extend a predicted distance of about 30 Å (32).

FokI is prevented from cleaving non-specific DNA strands by sequestering its C-terminal EN domain containing a single PD-(D/E)xK catalytic motif loosely against the N-terminal TRD (23,24). The TRD of FokI has an extra three α-helices compared to PaqCI that are used to dock the EN domain when FokI is not bound to a target site. These three additional α-helices in the TRD barely contact the DNA, implying that their primary role is to exclude the EN domain from the DNA (23,24). In contrast, PaqCI conserves the dimerization of the EN domains and sequesters each endonuclease dimer at the center of the quaternary TRD ring and away from solution.

In the FokI dimer, the TRDs face each other (DNA binding ‘C’ portion of the TRDs facing inward) instead of being back-to-back, as observed in the PaqCI tetramer. When bound, the double-stranded DNA substrates in FokI are, at their closest, ∼8 Å apart from each other, whereas PaqCI’s substrate is ∼50 Å apart (Supplemental Figure S6b). This difference potentially changes the orientation of the EN, TRD, and double-stranded DNA molecules in the cleavage dimer and may contribute to the distinctive cleavage distances and the variation in search mechanisms.

There appear to be few or no residues that establish base-specific contacts with the DNA in the EN domains in either PaqCI or FokI. Therefore, they likely rely on stereochemical constraints to define their cleavage sites and patterns relative to the bound target sites. Since the TRD is essential for DNA recognition and binding, we can hypothesize that the cleavage location is controlled by (i) the requirement for the EN dimer to re-associate around the cleavage site, coupled with (ii) the necessity that the *cis-*acting EN domain avoids steric clash with its own TRD and the *trans-*acting domain not be required to move beyond the reach of its linker peptide (23,25,26). This hypothesis is satisfactorily supported by an examination of the DNA-bound structure of PaqCI (for which it does not appear that the EN dimer can be positioned in an alternative location other than over the scissile phosphates). In contrast, it is more difficult to confidently assign a structural mechanism that would limit FokI to cleavage at a strongly preferred distance of 9 and 13 bases from its bound target site. Potentially, when the EN domain is released, the linker region becomes structured and might thereby act as a ‘measuring rod’, allowing the enzyme to cleave at 9 and 13 each time it binds its target site (26,32).

PaqCI and FokI both appear to generate a cleavage synapse with DNA in parallel arrangement (30–32). The binding of FokI to the DNA in parallel is due to the energetics of the loop formation on the same strand. This necessary looping is an interesting conundrum for PaqCI since it acts as a tetramer during target site binding and cleavage. Perhaps this is another mode of regulation to bias cleavage towards foreign DNA. In either case, the unwinding of the DNA post-cleavage likely upsets the binding of the EN domains and signals the enzyme to disengage and reengage a new cleavage site. Further experiments using atomic force spectroscopy and magnetic tweezers would be necessary to determine the specific DNA looping mechanism of PaqCI.

## DATA AVAILABILITY

The sequence of PaqCI is available as part of the deposited structure (PDB ID 8EM1 and 8EPX), which are also provided as supplementary material. The original source data and raw images corresponding to the biochemical analyses of PaqCI function and activity have been uploaded to the Harvard Dataverse public repository (https://dataverse.harvard.edu/dataverse/PaqCI). Permanent DOIs: 10.7910/DVN/2SZXFW, 10.7910/DVN/QEEZ7Y, 10.7910/DVN/TIHGAY, 10.7910/DVN/62YXDO, 10.7910/DVN/XTQZXI, 10.7910/DVN/5GHTIV, 10.7910/DVN/G8XG68. The crystallographic and cryoEM structures described in this manuscript have been deposited in the RCSB protein database (PDB ID codes: 8EM1 and 8EPX; EMDB ID code: EMD-28534).

## Supplementary Material

gkad228_Supplemental_Files

## Notes

Present address: Christopher J. Hosford, LifeMine Therapeutics, 30 Acorn Park Drive, Cambridge, MA 02140, USA.

Present address: Caleigh M. Azumaya, Genentech, 1 DNA Way, South San Francisco, CA 94080, USA.
