# Supplementary material for: Structures, activity and mechanism of the Type IIS restriction endonuclease PaqCI
Source: Nucleic Acids Res. 2023 Mar 29;51(9):4467–87. doi: 10.1093/nar/gkad228 (PMC10201449; doi:10.1093/nar/gkad228)

SUPPLEMENTARY INFORMATION

Structures, activity, and mechanism of the Type IIS restriction endonuclease PaqCI

Madison A. Kennedy[1], Christopher J. Hosford[2,3], Caleigh M. Azumaya[1,4], Yvette A. Luyten[2], Minyong Chen[2], Richard D. Morgan[2] and Barry L. Stoddard[1,#]

[1] Division of Basic Sciences
Fred Hutchinson Cancer Research Center
1100 Fairview Ave. North, Seattle WA 98109 USA

[2] New England Biolabs
240 County Road
Ipswich, MA 01938, USA

[3] Current Address:
LifeMine Therapeutics
30 Acorn Park Drive
Cambridge, MA 02140, USA

[4] Current Address:
Genentech
1 DNA Way
South San Francisco, CA 94080, USA

[#] Corresponding author (*bstoddar@fredhutch.org; 206-667-0431)*

Supplementary Table S1. Oligonucleotide primers

PaqCI_pUC19 1 For	CCT TTC GT**C ACC TGC** GTT TCG GTG ATG ACG GTG AA
PaqCI_pUC19 1 Rev	ACC GAA AC**G CAG GTG** ACG AAA GGG CCT CGT GAT ACG
PaqCI_pUC19 700HTT For	GCT TCC TCG **CAC CTG C**AC TCG CTG CGC TCG GTC
PaqCI_pUC19 700HTT Rev	GCA GCG AGT **GCA GGT G**CG AGG AAG CGG AAG AGC GCC
PaqCI_pUC19 700HTH For	GCT TCC TCG **GCA GGT G**AC TCG CTG CGC TCG GTC
PaqCI_pUC19 700HTH Rev	GCA GCG AGT **CAC CTG C**CG AGG AAG CGG AAG AGC GCC
PaqCI_pACYC184 camFOR	TAC T**GC AGG TG**C GAA GAG CAC TGG TGT CCC TGT TGA TAC CG
PaqCI_pACYC184 camREV	GTG A**GC AGG TG**C TGA GAC GAA CCA GGC GTT TAA GGG CAC CA
pUC19_addPaqCI_For	TGC TCT TCG **CAC CTG C**AG TAT ATA TGA GTA AAC TTG GTC TGA CAG TTA CCA
pUC19_addPaqCI_Rev	TCG TCT CAG **CAC CTG C**TC ACG TTC CAC TGA GCG TCA GAC C

*PaqCI recognition site in **BOLD** type
**HiFi assembly overlap underlined

SUPPLEMENTARY MOVIE CAPTIONS

Supplementary Movie S1. 3D variability analysis of the CryoEM DNA-free PaqCI.
A movie displaying the tetramer CryoEM map of DNA-free PaqCI showing an oscillation between either side of the dimer pair with correlated motions of each TRD affecting the corresponding orientation of its neighbor. 3D variability was calculated and compiled in cryoSPARC [43]. The movie was produced using UCSF Chimera [44]. This observation corresponds with what was seen in **Supplementary Figure S2e**, where the ends of the target recognition domains (TRDs) have the lowest resolution.

Supplementary Movie S2. 3D variability analysis of the CryoEM DNA-bound PaqCI.
A movie displaying the tetramer CryoEM map of DNA-bound PaqCI showing a lack of conformation sampling when in solution. The greatest variation in conformation is seen in the bound DNA duplexes, specifically the nucleotides furthest from the binding site. 3D variability was calculated and compiled in cryoSPARC [43]. The movie was produced using UCSF Chimera [44].

Supplementary Movie S3. Motion of the *cis* endonuclease when binding DNA.
A movie reflecting the motion of the *cis* endonuclease (EN) domain when its target recognition domain (TRD) binds to a target site. The EN domain is shown in pink. The TRD is shown in red. The other three monomers have been removed from this movie for clarity. Morph was made in Pymol using the x-ray crystal structure of the DNA-free PaqCI and the CryoEM structure of the DNA-bound PaqCI.

Supplementary Movie S4. Motion of the *trans* endonuclease when binding DNA.
A movie reflecting the large motion of the *trans* endonuclease (EN) domain when its target recognition domain (TRD) binds to a target site. The *cis* monomer and the DNA are represented for clarity in the movie but are not moving. The *trans* EN domain is shown in light green and its TRD in dark green. The *cis* EN domain is shown in pink and its TRD in red. The other two monomers have been removed from this movie for clarity. Morph was made in Pymol using the x-ray crystal structure of the DNA-free PaqCI and the CryoEM structure of the DNA-bound PaqCI.

SUPPLEMENTARY FIGURE CAPTIONS

Figure S1. Purification and solution behavior of PaqCI.

Panel a: SDS-PAGE gel of induced cell lysate and purification via metal affinity chromatography.

Panel b: Size exclusion chromatographic elution of final purified PaqCI used for subsequent structural analyses. The protein elution profile is dark blue; underlying elution of molecular weight standards is light dashed grey. The expected peak elution volumes for an enzyme monomer (56 kD) or dimer (112 kD) are indicated with labeled arrows. The enzyme elutes as a multimeric complex that is consistent with an enzyme tetramer.

Figure S2. Crystallographic and CryoEM analysis of DNA-free PaqCI apo-enzyme.

Panel a: Crystals and diffraction pattern for DNA-free PaqCI. Diffraction pattern corresponds to a 0.5 second, half-degree oscillation collected at the Advance Light Source (ALS) beamline 5.0.1 on a Pilatus area detector.

Panel b: Ribbon diagram and corresponding electrostatic surface of the DNA-free PaqCI tetramer. The enzyme complex does not present extensive regions of strongly basic, positively charged surface area, corresponding to a lower calculated pI (approximately 8.0) that necessitated formation of a stable DNA complex at neutral pH.

Panel c: Representative negative stain micrograph and class averaged 2D particle density for DNA-free PaqCI.

Panel d: Representative CryoEM micrograph and class averaged 2D particle density for DNA-free PaqCI.

Panel e: The 3D CryoEM reconstruction of DNA-free PaqCI as calculated in cryoSPARC. The image of the map was generated in UCSF Chimera.

Figure S3. CryoEM analysis of PaqCI DNA-bound enzyme.

Panel a: Size exclusion chromatographic elution behavior of PaqCI enzyme in the presence of a stoichiometric excess of double-strand DNA (50 basepair DNA shown in **Figure 4a**) indicates formation of a stable DNA-bound enzyme complex. The elution profiles for free protein, free DNA and a 1:1.2 stoichiometric mixture of protein and DNA are shown as bold colored lines; the elution of molecular weight standards is shown as a light dashed grey profile The expected peak elution volumes for an enzyme monomer (56 kD) or dimer (112 kD) are indicated with labeled arrows. In this experiment, the DNA (and the protein-DNA complex) elute at a significant early volume due to the length of the DNA duplex used in the experiments.

Panel b: Class averaged 2D images of DNA-bound PaqCI complexes. The bound DNA molecules extending from the protein assemblage are readily visible.

Panel c: CryoEM electron density for the regions of the *cis*-acting and *trans*-acting endonuclease domains in complex with their respective scissile phosphate groups on each DNA strand of the enzyme's cleavage site. A single-bound calcium ion is associated with a non-bridging oxygen of each scissile phosphate and a catalytic aspartate residue of each endonuclease domain.

Figure S4: CryoEM analysis approach for DNA-bound PaqCI.

Panel a: The flowchart for CryoEM data processing. Heterogeneous structures in figure represent only the 40° tilt series (since this step was conducted before combining the data sets) but was repeated with the 0° tilt series. Chosen structures from the heterogeneous jobs are highlighted with a black box. Details can be found in Methods.

Panel b: The 3D CryoEM reconstruction of DNA-bound PaqCI as calculated in cryoSPARC. The image of the map was generated in UCSF Chimera.

Panel c: Fourier shell correlation (FSC) curve for the 3D reconstruction of DNA-bound PaqCI from cryoSPARC.

Supplemental Figure S5. DNA binding by individual subunits of the PaqCI tetramer.

Panel a: Side by side comparison and superposition of the four target recognition domains (TRDs) in the absence and presence of bound DNA indicates minimal rearrangement of the tetrameric assemblage of those domains.

Panel b: Contact maps (generated using the DNAProDB online analysis tool [45]) for two protein subunits (A and D, *trans* and *cis*) that complete the DNA-bound dimer-of-dimer of the enzyme tetramer in complex with independently bound DNA duplexes. The associated TRD and EN domains are boxed.

Supplemental Figure S6: Comparison of FokI and PaqCI structural organization, activity, and DNA complexes.

Panel a: Domain organization, target sites and cleavage patterns of FokI (left) and PaqCI (right)

Panel b: Comparison of DNA-bound dimers visualized via single particle electron microscopy for FokI (left; figure panel figure panel generated from [32] and from the model provided by the authors) and PaqCI (right; this study). The latter enzyme-DNA complex is extracted from the larger DNA-bound PaqCI tetramer and corresponds to the dimer in that assemblage that displays visible DNA-engaged endonuclease domains.

Figure S1

a

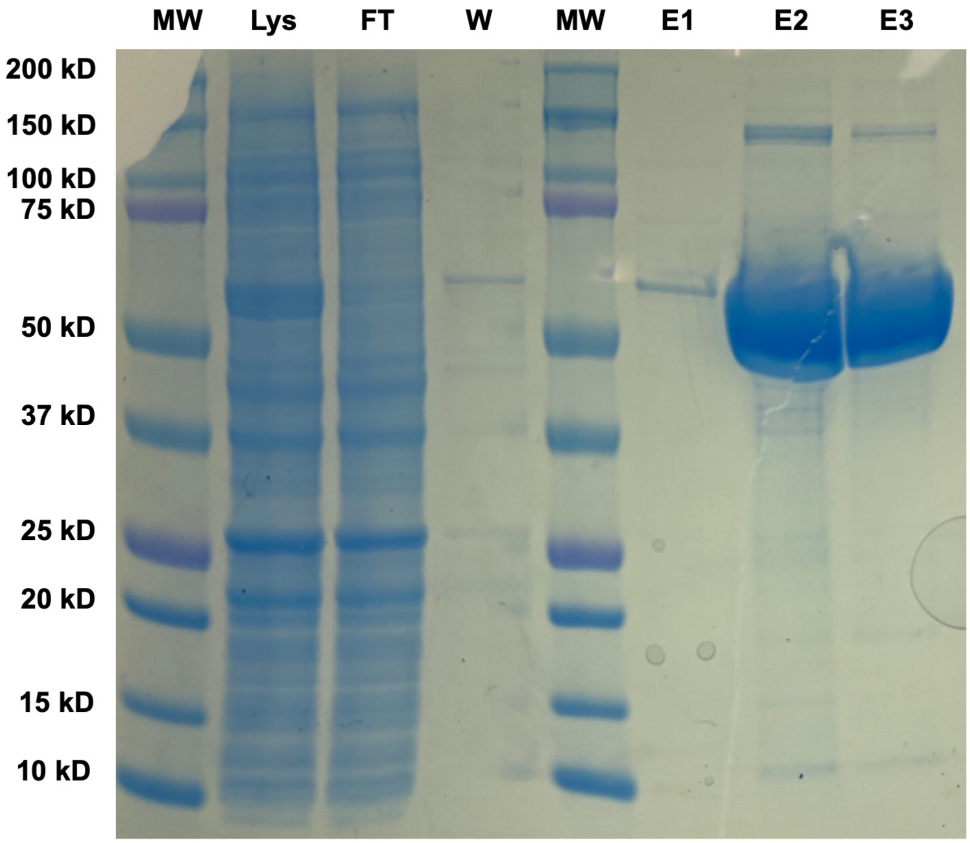

MW
Lys
FT
W
MW
E1
E2
E3
200 kD
150 kD
100 kD
75 kD
50 kD
37 kD
25 kD
20 kD
15 kD
10 kD

b

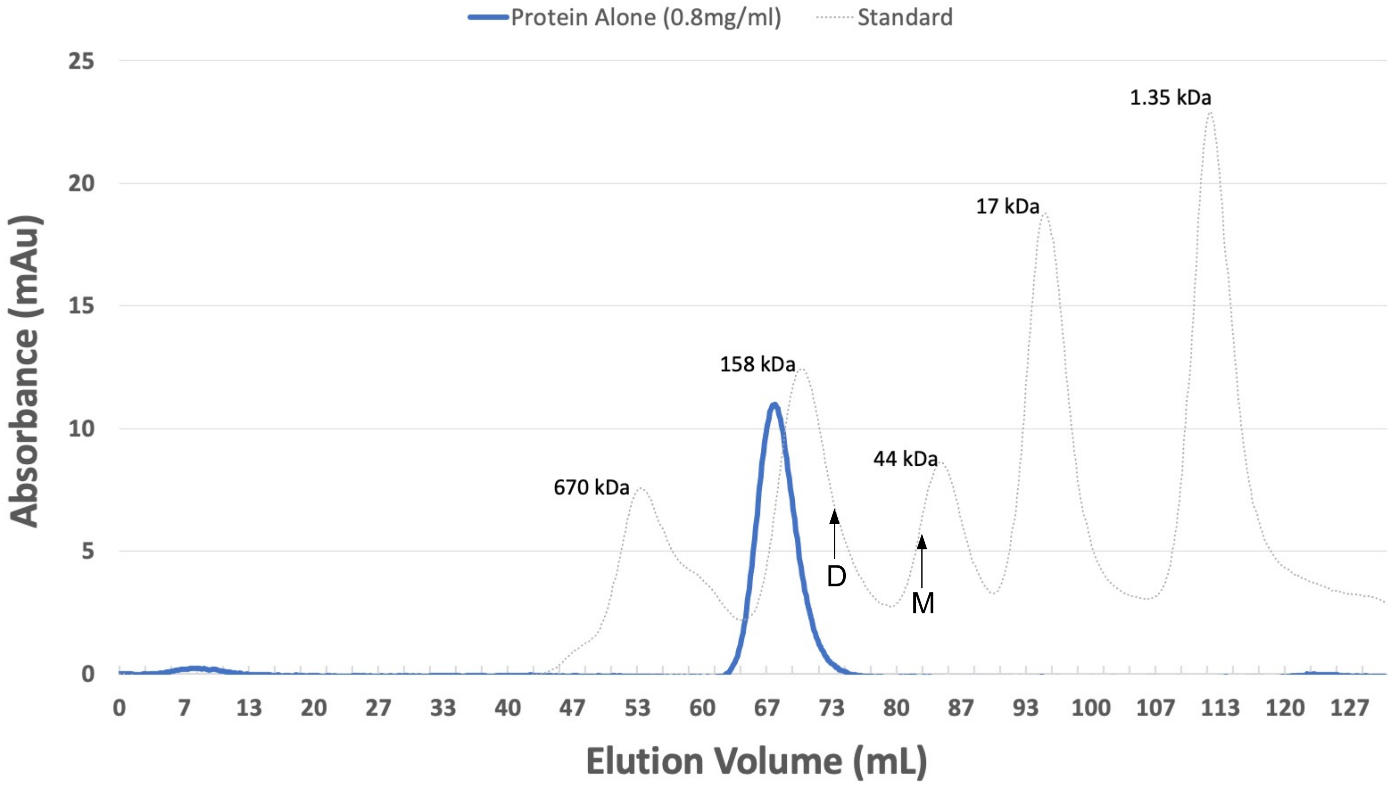

Protein Alone (0.8mg/ml)
Standard
Absorbance (mAu)
Elution Volume (mL)
0
5
10
15
20
25
0 7 13 20 27 33 40 47 53 60 67 73 80 87 93 100 107 113 120 127
670 kDa
158 kDa
44 kDa
17 kDa
1.35 kDa
D
M

Figure S2

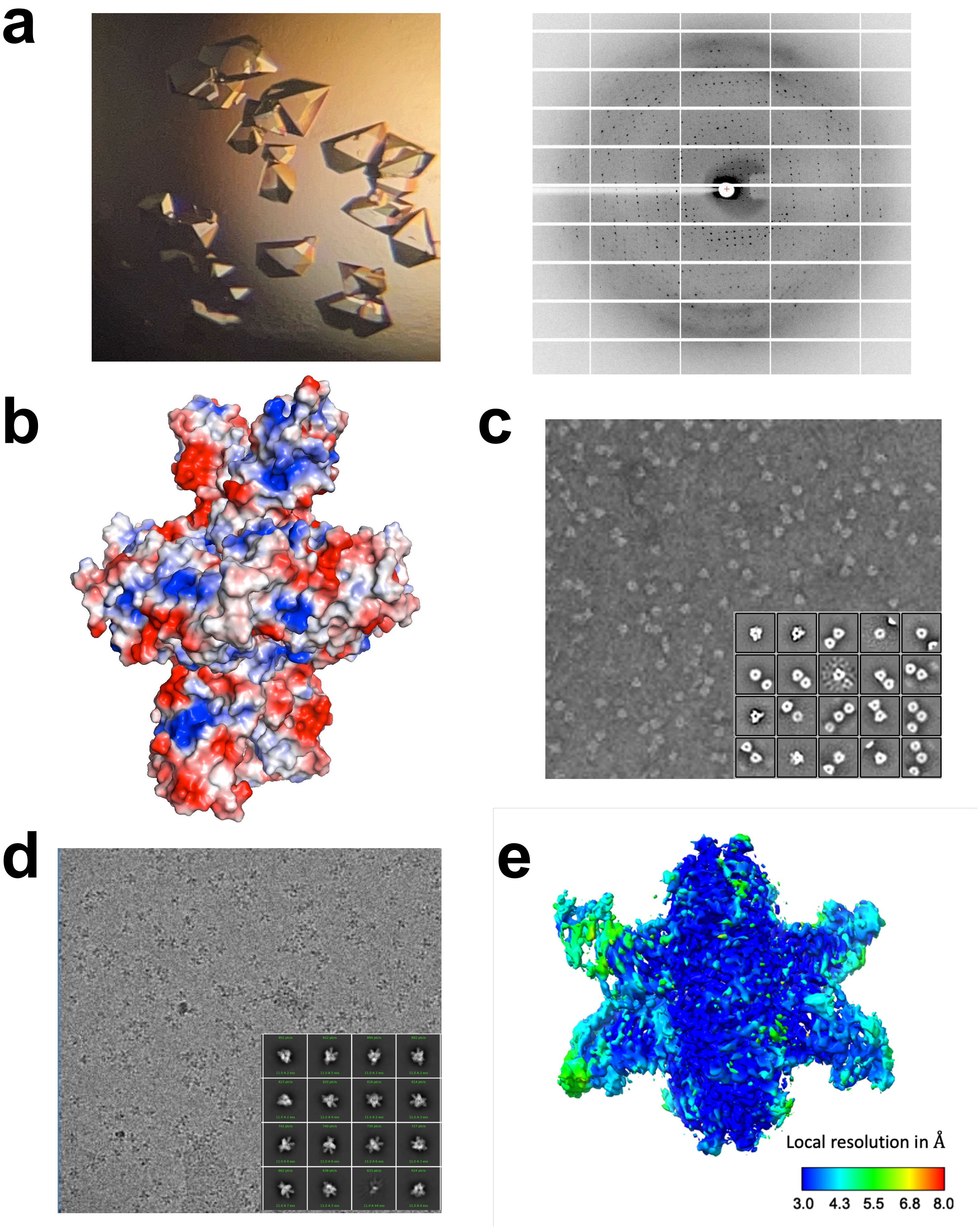

Figure S3

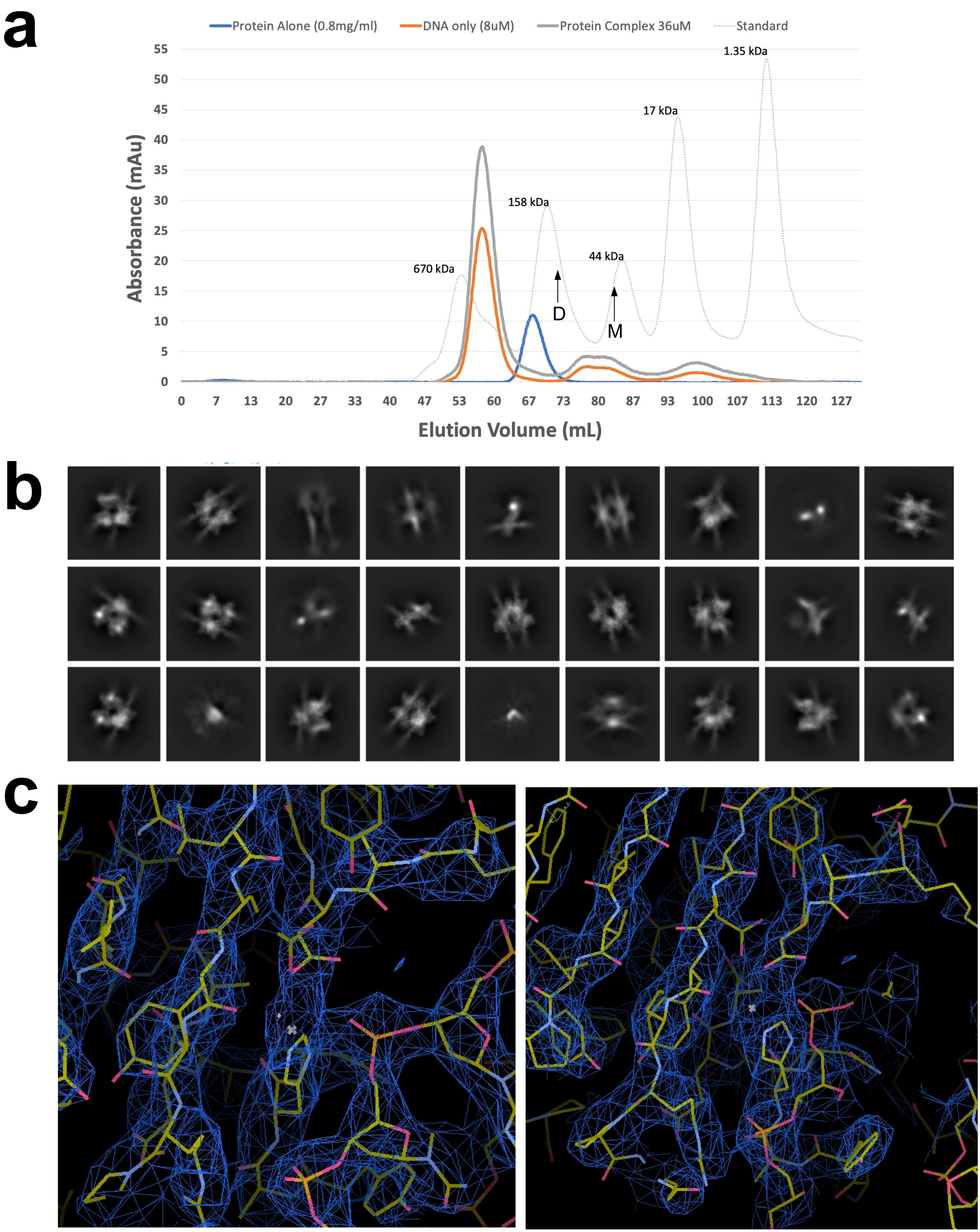

cis-acting cleavage site

trans-acting cleavage site

Figure S4

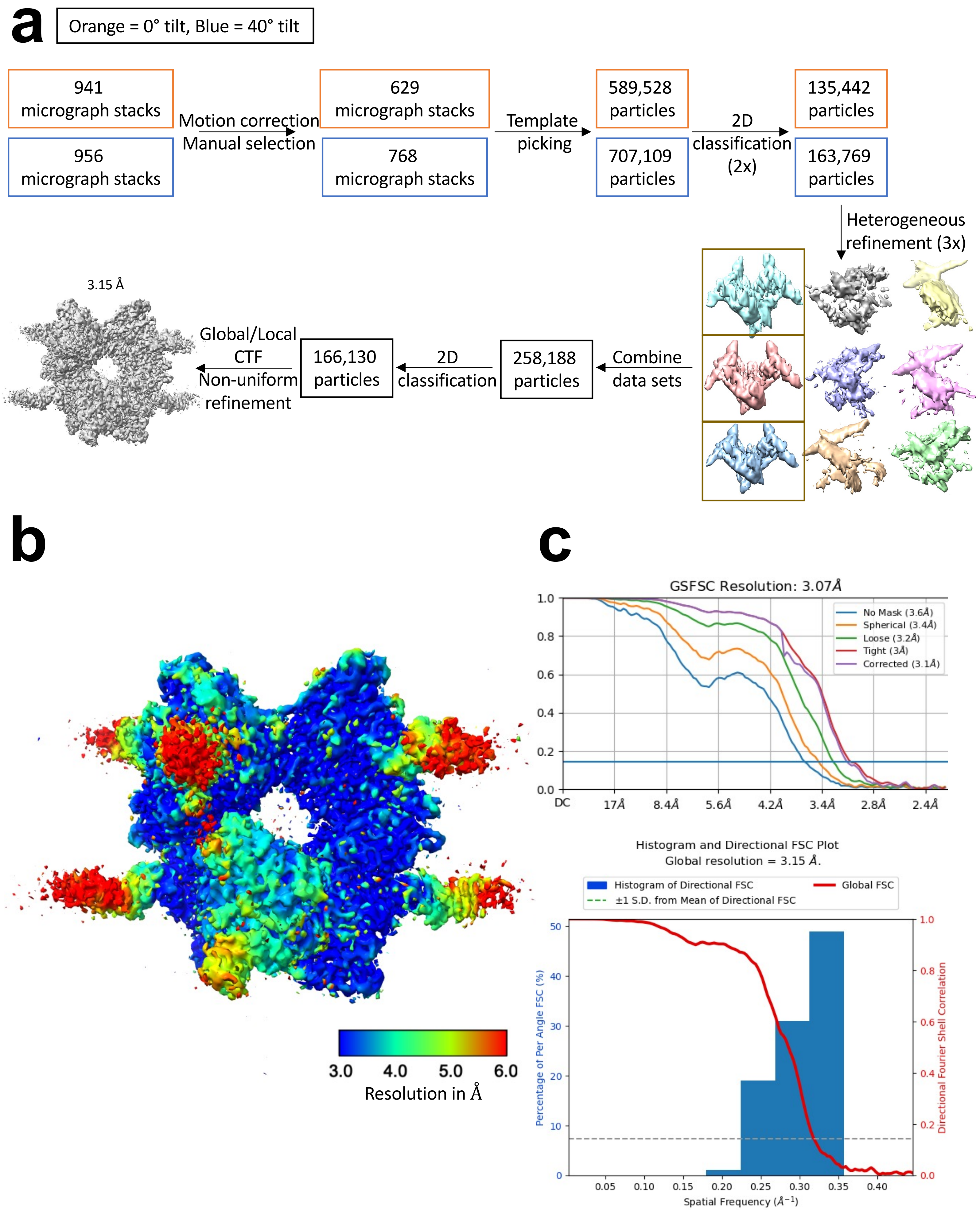
a
Orange = 0° tilt, Blue = 40° tilt
941 micrograph stacks
956 micrograph stacks
Motion correction
Manual selection
629 micrograph stacks
768 micrograph stacks
Template picking
589,528 particles
707,109 particles
2D classification (2x)
135,442 particles
163,769 particles
Heterogeneous refinement (3x)
Combine data sets
258,188 particles
2D classification
166,130 particles
Global/Local CTF
Non-uniform refinement
3.15 Å
b
3.0
4.0
5.0
6.0
Resolution in Å
c
GSFSC Resolution: 3.07Å
No Mask (3.6Å)
Spherical (3.4Å)
Loose (3.2Å)
Tight (3Å)
Corrected (3.1Å)
DC
17Å
8.4Å
5.6Å
4.2Å
3.4Å
2.8Å
2.4Å
Histogram and Directional FSC Plot
Global resolution = 3.15 Å.
Histogram of Directional FSC
Global FSC
±1 S.D. from Mean of Directional FSC
Percentage of Per Angle FSC (%)
Directional Fourier Shell Correlation
Spatial Frequency (Å⁻¹)

Figure S5

a

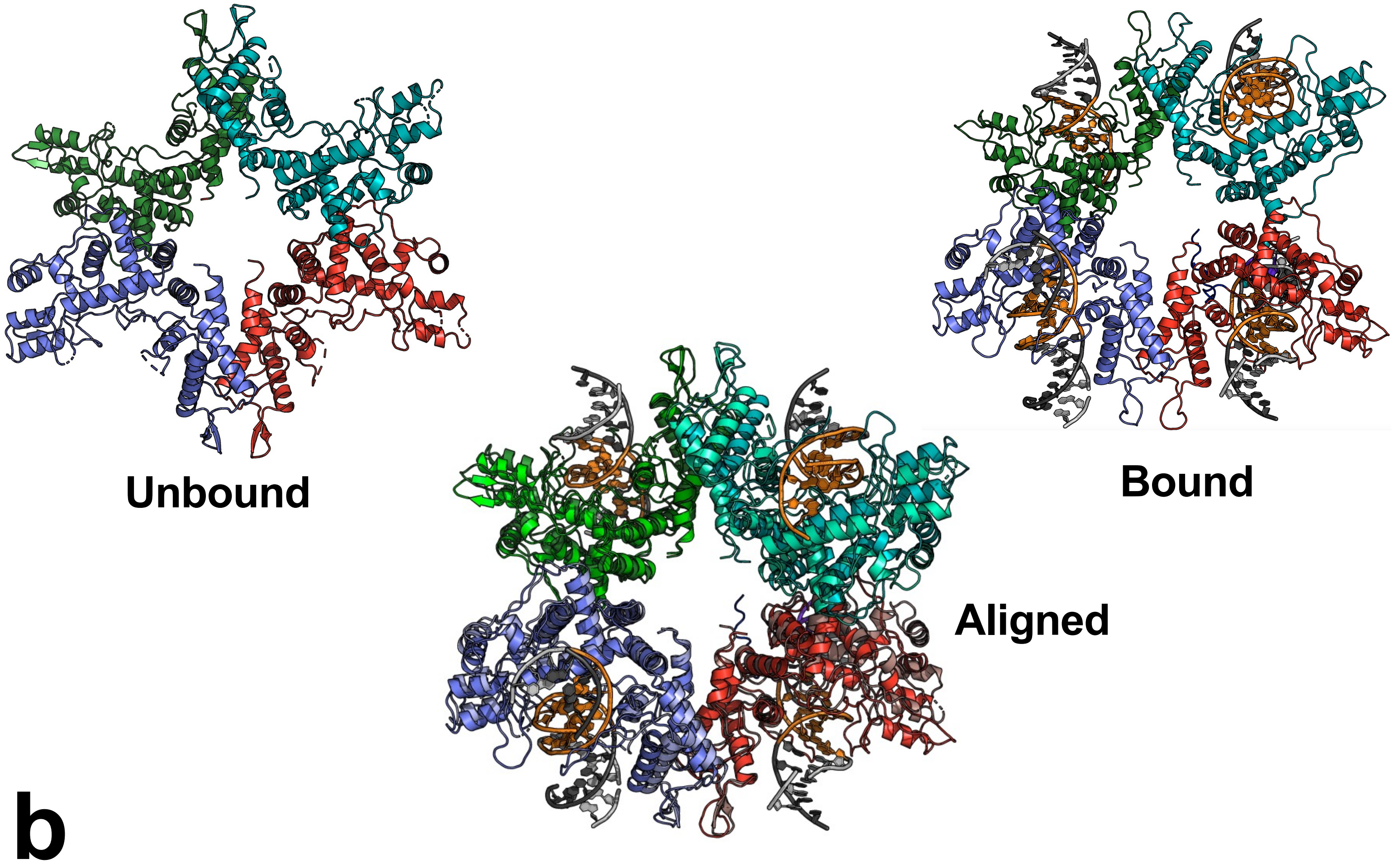

Unbound
Bound
Aligned

b

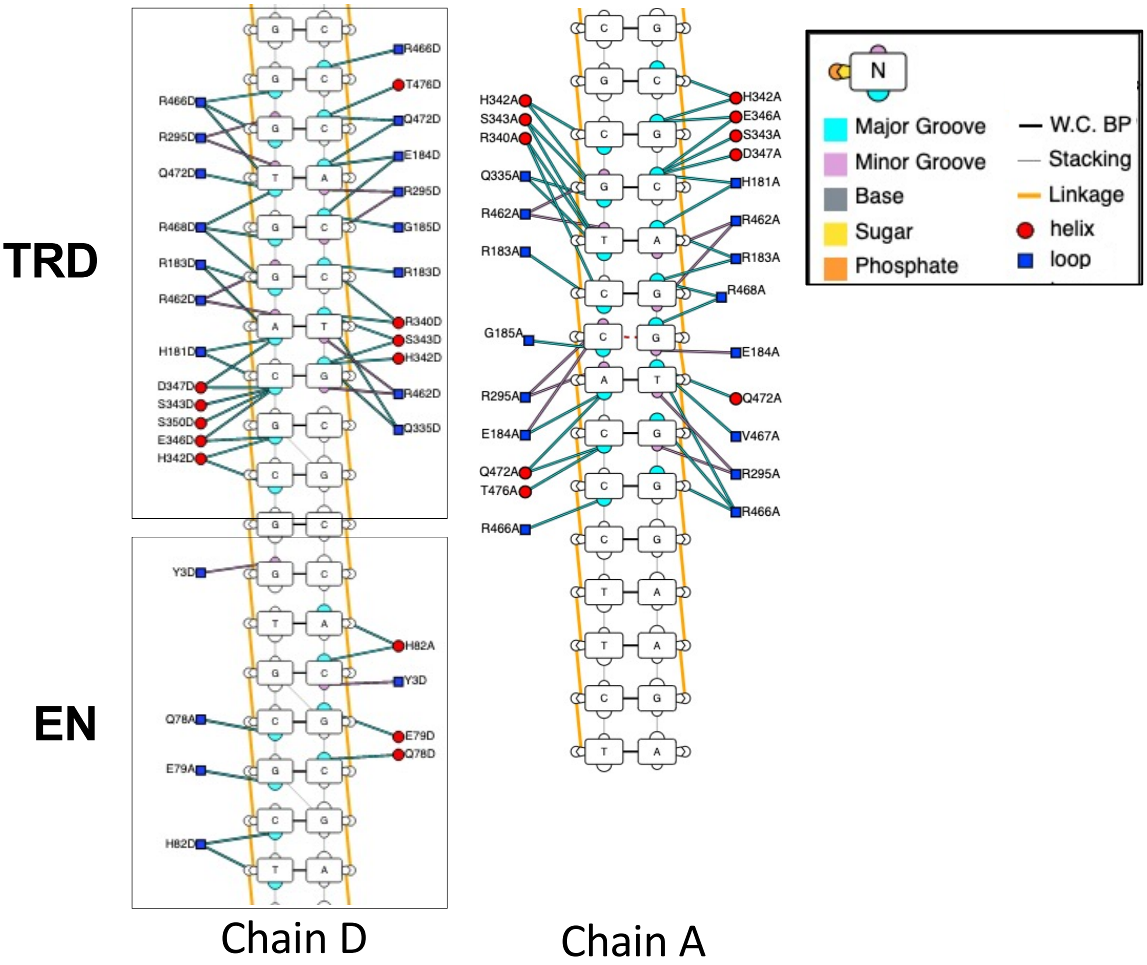

TRD
EN
N
Major Groove
Minor Groove
Base
Sugar
Phosphate
W.C. BP
Stacking
Linkage
helix
loop
Chain D
Chain A

Figure S6

a

FokI

- PD-(D/E)xK
- 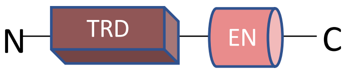

- 5′ GGATGNNNNNNNNNN| NNNNN 3′
  3′ CCTACNNNNNNNNNNNNNN| N 5′

PaqCI

- PD-(D/E)xK
- 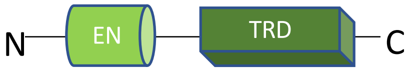

- 5′ CACCTGCNNNN| NNNNN 3′
  3′ GTGGACGNNNNNNNNN| N 5′

b

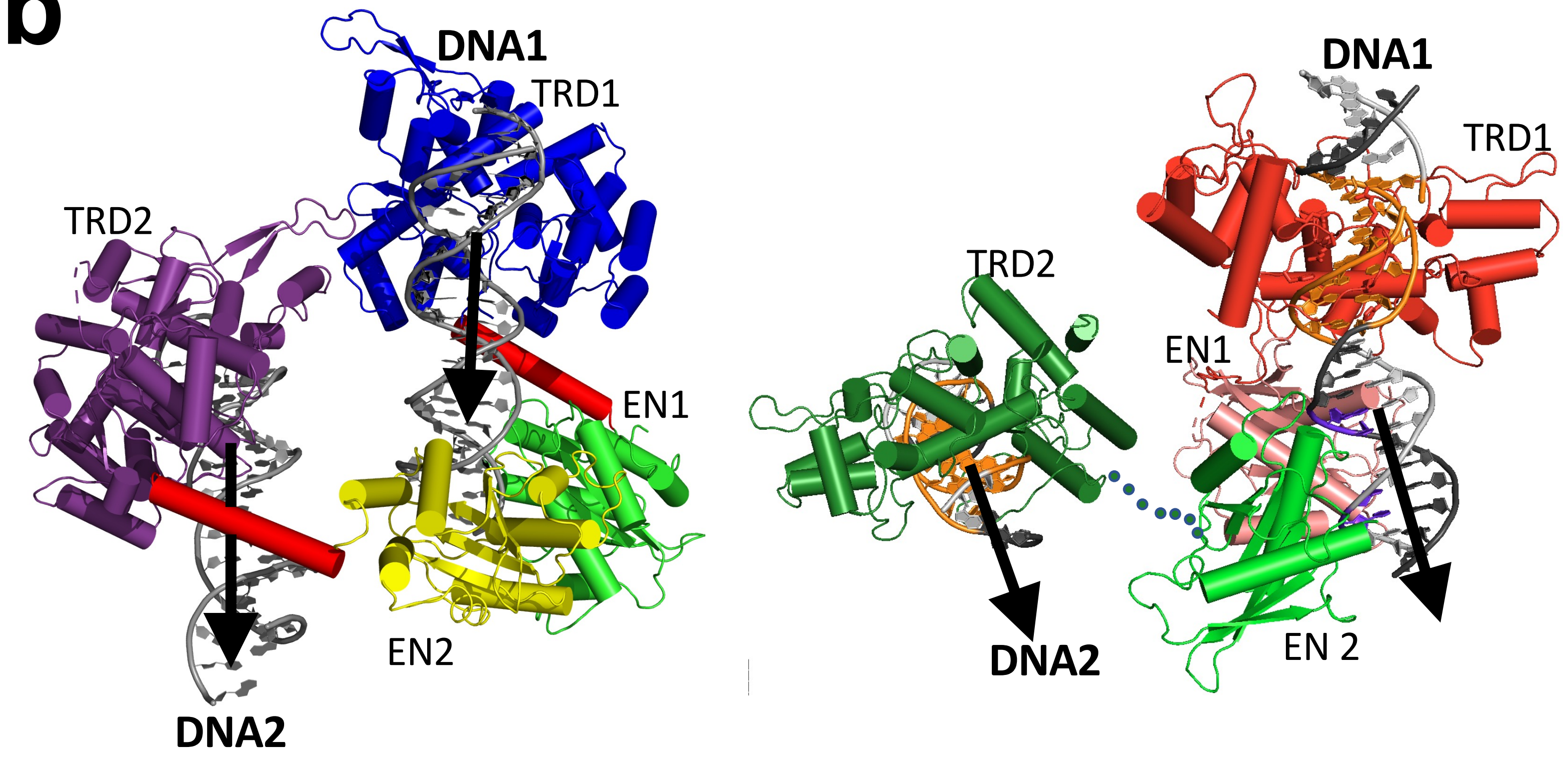

Supplement: gkad228_Supplemental_Files [file gkad228_supplemental_files.zip › SUPPLEMENTARY INFORMATION_FINAL_27Jan2023.pdf]
